# Human mediated translocation of Pacific paper mulberry [*Broussonetia papyrifera* (L.) L’Hér. ex Vent. (Moraceae)]: Genetic evidence of dispersal routes in Remote Oceania

**DOI:** 10.1371/journal.pone.0217107

**Published:** 2019-06-19

**Authors:** Gabriela Olivares, Bárbara Peña-Ahumada, Johany Peñailillo, Claudia Payacán, Ximena Moncada, Mónica Saldarriaga-Córdoba, Elizabeth Matisoo-Smith, Kuo-Fang Chung, Daniela Seelenfreund, Andrea Seelenfreund

**Affiliations:** 1 Department of Biochemistry and Molecular Biology, Facultad de Ciencias Químicas y Farmacéuticas, Universidad de Chile, Santiago, Chile; 2 Centro de Investigación en Recursos Naturales y Sustentabilidad, Universidad Bernardo O’Higgins, Santiago, Chile; 3 Department of Anatomy, University of Otago, Dunedin, New Zealand; 4 Research Museum and Herbarium (HAST), Biodiversity Research Center, Academia Sinica, Taipei, Taiwan; 5 Escuela de Antropología, Facultad de Ciencias Sociales, Universidad Academia de Humanismo Cristiano, Santiago, Chile; Missouri Botanical Garden, UNITED STATES

## Abstract

Paper mulberry, *Broussonetia papyrifera* (L.) L’Hér. ex Vent. (Moraceae), a dioecious species, was transported by humans from Taiwan to the islands of Remote Oceania. Its introduction and cultivation in Remote Oceania was intentional due to its cultural importance as a fiber source for barkcloth textiles. The aim of this study was to explore the genetic diversity and structure of paper mulberry populations within Remote Oceania in order to infer dispersal patterns that may reflect past human interaction among island groups. We present the integrated analysis of 380 samples (313 contemporary and 67 herbarium specimens) collected in Near and Remote Oceania. Genetic characterization was based on a set of ten microsatellites developed for *B*. *papyrifera* and complemented with the analysis of the ribosomal internal transcribed spacer ITS-1 sequence, a sex marker and the chloroplast *ndhF–rpl32* intergenic spacer. Microsatellite data identify a total of 64 genotypes, despite this being a clonally propagated crop, and show three major dispersal hubs within Remote Oceania, centered on the islands of Fiji, Tonga, and Pitcairn. Of 64 genotypes identified, 55 correspond to genotypes associated to female-sexed plants that probably descend from plants introduced by the prehistoric Austronesian-speaking voyagers. The ratio of accessions to genotypes between herbarium and contemporary samples, suggests recent loss of genetic diversity. In addition to the chloroplast haplotypes described previously, we detected two new haplotypes within Remote Oceania both originating in Taiwan. This is the first study of a commensal species to show genetic structuring within Remote Oceania. In spite of the genetic bottleneck, the presence of only one sex, a timespan of less than 5000 years, and asexual propagation of this crop in Remote Oceania, we detect genetic diversity and regional structuring. These observations suggest specific migration routes between island groups within Remote Oceania.

## Introduction

The last stage of human colonization of uninhabited regions of our planet, involving the settlement of the remote islands in the Pacific Ocean, has fascinated many, from early European explorers to historians, archaeologists, and, more recently, geneticists and Pacific communities. Archaeological research has allowed for the construction of a chronological framework for the main migratory waves and associated cultural history [1]. The human settlement of this region began over 45,000 years ago with the arrival of the first populations to New Guinea, New Britain, New Ireland and the Solomon Islands [2]. This region is known as Near Oceania. The region extending to the east of the Solomon Islands, known as Remote Oceania, began to be settled much more recently [3]. Western Remote Oceania (WRO), which includes New Caledonia, Vanuatu, Fiji, Wallis, Samoa, Tonga and some of the islands of Micronesia, was settled between 3000 and 2800 years before present (BP). The islands of Eastern Remote Oceania (ERO), including the Society, Marquesas, Cook and Austral Islands, as well as the Hawaiian archipelago, New Zealand and Rapa Nui (Easter Island), were not settled until at least 1500 years later, beginning around 1000 years BP [1, 4].

While linguistic studies have determined that the origin of the Austronesian languages spoken in the Pacific region is in Taiwan [5], genetic studies have revealed the complexities of this human migratory process [2]. Colonization of these new environments involved the transport and introduction of a range of animal and plant species from mainland Asia, Island Southeast Asia and New Guinea to ensure survival of the settlers in the increasingly distant and terrestrial resource-depauperate islands [2]. The study of human movements using plant or animal species closely associated to humans as a proxy is known as the ‘commensal approach’. It applies to the study of species introduced exclusively by people in the past, to geographic regions beyond their natural habitat [6, 7, 8, 9]. However, some animal commensal species such as dogs and pigs were not introduced into all islands and in general were not as widely dispersed as plant crops; other species such as chickens have not been studied in the complete introduced range, specially not in the eastern regions of Remote Oceania. Important crops like taro (*Colocasia esculenta*) [10], bananas (*Musa* spp.) [11, 12, 13], breadfruit (*Artocarpus* spp) [14] yams (*Dioscorea* spp) [15], kava (*Piper methisticum*) [16] and sweet potato *(Ipomea batatas)* [17] have been studied using the commensal approach, revealing multiple origins of these species. Paper mulberry (*Broussonetia papyrifera* (L.) L’Hér. ex Vent. (Moraceae)), a dioecious plant native to China, Taiwan and Indochina [18], where it was used as source of fiber for making high quality paper and for medicinal purposes [19], was also introduced by the early voyagers, as an important part of Austronesian material culture for making barkcloth textiles and cordage [20, 21]. Paper mulberry was introduced into all Pacific islands, as far to the east as Rapa Nui. *B*. *papyrifera* is reproduced asexually in the Pacific islands, a common practice for many crops in Oceanian arbori- and vegeculture [22]. Its single sex nature in the Pacific prevents the production of seeds and natural dispersal within and between islands [21].

We have previously analyzed contemporary specimens of *B*. *papyrifera*, using different molecular markers, to infer human movements in Oceania. Using the internal transcribed spacer (ITS) ribosomal region, it is possible to distinguish between Asian and Oceanian samples through a single nucleotide polymorphism [23, 24]. Analyses with multilocus inter-simple sequence repeat (ISSR) markers [24] suggest a prehistoric human-mediated introduction of paper mulberry from East Asia to Remote Oceania and a second human-mediated introduction to Hawaii, probably in historic times. This is supported by data obtained using a sex maker [25] that indicates the presence of only female individuals of paper mulberry in Remote Oceania, with the exception of Hawaii, where male plants are also found [25]. Recent studies using the *ndh-rpl32* hypervariable chloroplast DNA marker showed that the most common haplotype (cp17) of paper mulberry found in the Pacific islands from New Guinea to Easter Island, and the most likely to have been introduced by the early colonizers, showed a tight phylogeographic link to Taiwan, providing the first genetic proof based on plants of the “Out of Taiwan” hypothesis of the Austronesian expansion [26]. Also, using microsatellites on a limited set of paper mulberry herbarium samples, we were able to detect genetic diversity within Near and Remote Oceania, in spite of the vegetative propagation of this species in Oceania. Genotypes were structured into two major groups, separating West and East Remote Oceania and placing Pitcairn in a pivotal position between these regions, suggesting pulses during the dispersal history [27]. A fundamental question that remained unanswered with the previous studies is whether the genetic diversity of paper mulberry detected with microsatellites within Remote Oceania can be used as an effective proxy of past human movements. To answer this question, we characterized an enlarged set of samples of both contemporary plants and herbarium specimens. The aim of this study was to explore the genetic diversity and structure of paper mulberry populations in order to infer past human-mediated translocations of this species to reveal dispersal patterns among islands within Remote Oceania.

Here, we report the combined analysis of a total of 380 samples (313 contemporary and 67 herbarium accessions) based on the ITS-1 region [23, 24], a sex marker [25], the *ndh-rpl32* cpDNA region [26] and nine microsatellite markers [27] previously used by our group.

The use of microsatellites is warranted because of their abundancy, robustness, high reproducibility, codominant nature and randomly dispersed polymorphisms in the genome. Therefore, they are efficient markers for population genetic studies [28, 29, 30]. We analyzed the genetic diversity of contemporary paper mulberry as seen with microsatellites and compared this diversity with the published and new data of herbarium samples. Our results allow us to infer patterns of human mobility and migration across the Pacific region.

## Materials and methods

### Collection of samples

A total of 380 specimens, of which 313 correspond to contemporary plants and 67 to herbarium specimens, were analyzed. Samples were collected from 39 different localities, 34 from Pacific islands, four from the native range in Asia, and one from South America (S1 Table, S1 Fig). A total of 158 contemporary specimens collected between 2008 and 2016 under projects FONDECYT 1080061 and 1120175 were obtained from New Caledonia, Fiji, Samoa, Wallis and Tonga in West Remote Oceania (WRO); 121 were collected from East Remote Oceania (ERO) locations (Hawaii, Tahiti, the Marquesas Archipelago, Rapa in the Austral Islands, Rapa Nui and Pitcairn); 34 samples were collected from China, Taiwan and Vietnam, all in the native range, and from Japan; with one sample from South America (Santiago de Chile). Herbarium samples were taken from 67 specimens (S2 Table)] collected between 1882 and 2006 following the protocol designed by Payacán et al. [27]. Samples were provided by the following Institutions: Herbarium Pacificum (BISH), B.P. Bishop Museum in Honolulu, Hawaii, USA; Herbario Nacional (SGO), National Museum of Natural History in Santiago, Chile and the Auckland Museum Herbarium (AK) at the Auckland War Memorial Museum, Auckland, New Zealand. Sampling permits were obtained from relevant authorities when applicable (see S1 Text for complete list). In some cases, no official permits were issued (Fiji, Tonga, Samoa). In these cases, field owners and/or village chiefs gave oral permission to sample their fields after conversations explaining our project. We obtained all required permits and approvals as apply to foreign researchers at research sites where formal permission and approval were required. Details are given in the supplementary text (S1 Text). Samples from extant plants are kept at Universidad de Chile while this research continues.

### DNA extraction

Genomic DNA from contemporary samples was extracted following a CTAB extraction protocol as reported previously [23, 31, 27] based on Lodhi et al. [32]. The herbarium sample extractions were carried out in the ancient DNA laboratory of the University of Otago, following stringent ancient DNA contamination control protocols as described in Payacán et al. [27]. DNA quality from all samples was determined by the 260 nm/280 nm absorbance ratio using Nanodrop 2000 and DNA concentration was determined by fluorescence using the Quant-iTtM PicoGreen dsDNA Assay Kit (#7589) as indicated by the provider (Thermo Fisher Scientific). Integrity of extracted DNA was assessed by electrophoresis on 0.8% agarose gels.

### Amplification and genotyping

Samples were analyzed using four different molecular markers: i) the nuclear ITS-1 region, following the protocol described by González-Lorca et al. [24]; ii) the sex marker of 420 and 273 bp, as described by Peñailillo et al. [25]. For degraded DNA, specific primer pairs (300 and 165 bp) were designed for the amplification of a smaller region of the sex marker; iii) the *ndhF-rpl32* chloroplast DNA region using the primers and protocol as described in Chang et al. [26] with a subset of 35 contemporary samples representative of the islands sampled, and iv) a set of four nuclear microsatellite or Single Sequence Repeat (SSR) markers (Bro 07, Bro 08, Bro 13 and Bro 15) developed by one of us (K-FC, S3 Table) and six nuclear microsatellite markers for *B*. *papyrifera* (Bropap 2214, Bropap 2801, Bropap 20558, Bropap 25444, Bropap 26985 and Bropap 30248) as described in Peñailillo et al. [33]. Primers used in this study are presented in S3 Table. Herbarium samples were amplified following the protocols described by Payacán et al. [27], using reagents and materials exclusive for work with our herbarium DNA samples.

### Data analysis

ITS-1 and *ndhF-rpl32* regions. The amplified samples were sequenced at Macrogen Inc. (Seoul, South Korea). Electropherograms were checked using BioEdit 7.1.3.0 software [34] and polymorphisms were determined by sequence alignment using the Clustal W algorithm [35] in CLC Sequence Viewer software, as described [24, 27]. The ITS-1 sequences obtained from the herbarium collection were analyzed using the NCBI BLASTn tool (https://blast.ncbi.nlm.nih.gov) and identified as *B*. *papyrifera* to avoid possible misidentifications [27]. All *B*. *papyrifera* ITS-1 sequences were analyzed for the single nucleotide polymorphism (SNP) located at position 203 previously identified by our group [23, 24, 27].

#### Sex marker

The amplified samples were analyzed by visual inspection using 1.5% agarose gels. As described in Peñailillo et al. [25], female samples displayed a single 420 bp band, while male samples exhibited two bands at 273 and 420 bp.

#### cpDNA data

cpDNA haplotypes using the *ndhF-rpl32* region were obtained as described by Chang et al. [26]. Alignment of genetic data was performed with BioEdit software version 7.1.3.0 [36] and confirmed with GeneDoc version 2.7000 [37].

#### Microsatellites

The amplified samples were analyzed by capillary electrophoresis at the sequencing services from the Pontificia Universidad Católica de Chile (Santiago, Chile) and electropherograms were visualized and analyzed with Peak Scanner v1.0 software (Applied Biosystems). Allele sizes were registered in an Excel spreadsheet, as described by Payacán et al. [27] and allele frequency and genotypes were determined using GenAlex v.6.503 software [38].

In order to establish the number of real populations and the genetic admixture in the data obtained from the microsatellite markers, we performed a Bayesian population structure analysis using the Structure v 2.3.4 software [39]. To determine the most appropriate number of populations (*K*), a burn-in period of 5,000 was used in ten independent runs, and data were collected over 50,000 Markov Chain Monte Carlo (MCMC) replications from *K* = 1 to *K* = 15. The probability values were averaged across runs for each cluster. This procedure clusters individuals into populations and estimates the proportion of membership in each population for each individual [39]. The *K* value was determined by the log probability of data (Ln P(D)) based on the rate of change in LnP(D) between successive *K* values. The optimum *K* value was predicted following the simulation method of Evanno et al. 2005 [40] using the web-based software STRUCTURE HARVESTER version 0.6.92 [41] with a burn-in period of 500,000 in 20 independent runs, and data were collected over 750,000 Markov Chain Monte Carlo (MCMC) replications from *K* = 1 to *K* = 8.

To determine the relationships among genotypes, we constructed a Minimum Spanning Tree (MST) using BioNumerics v.7.6 (Applied Maths NV) as described Payacán et al. [27] using the genotypes identified in the introduced range. A genotype matrix identified within Remote Oceania and New Guinea was constructed by islands (e.g. Rapa Nui, Pitcairn), archipelagos or island groups (e.g. Marquesas, Samoa or Tonga). Genotypes found in New Zealand, Solomon Islands and Santiago were excluded. The similarity matrix was calculated using the categorical coefficient. Priority rules 1 and 2 were used with maximum number of N-locus variants (N = 1), weight: 10,000 and maximum number of N-locus variants (N = 2), weight: 10, respectively.

A second network was built using EDENetworks [42] to construct an MST based on the genotype matrix, diploid and sampling sites. The matrix was constructed with 380 samples, which were separated by localities corresponding to individual islands (and not to archipelagos or island groups) generating 45 sampling points. In the case of Hawaii, samples were separated by male and female specimens, and also a third group of individuals that could not be sexed; for this reason, the network was generated with 47 nodes. An MST is the minimal network necessary to connect all genotypes collected at different geographic locations (sites) within the data set. For this purpose, the program plots all sites (nodes) in a network graph with connections (edges) between all nodes. Each edge was weighted according to its pairwise genetic Goldstein genetic distance measure (δμ2) [34]. The MST selects a subset of edges that connects all nodes while minimizing the overall genetic distance. The layout of the MST was recalculated 1000 times to generate a 50% bootstrap tree. The resulting network was then manually manipulated (without changing the degree of connectedness) to better conform to geographic reality.

## Results

### Genetic analysis of contemporary and herbarium samples with rDNA, cpDNA and sex markers

Genomic DNA was successfully extracted from all contemporary leaf and herbarium samples using the Lodhi protocol [32] modified as indicated in the samples and methods section. All 380 samples (S1 and S2 Tables) were successfully amplified with the ITS-1 marker. A total of 292 (261 contemporary and 31 herbarium) samples presented the transversion from G to T (g. 203G>T) and 62 samples (52 contemporary and ten herbarium) carried the variant G. Twenty-three herbarium samples presented a double signal at this position, and in three samples this polymorphic position could not be read. A new SNP, a C to T transition at the relative position 99 (g. 99C>T), was detected and successfully analyzed in the 313 contemporary samples, revealing that 290 samples presented the C wild type (Asian) variant and 23 samples (18 samples from Hawaii, three from Vietnam and two from China) presented the T variant at this position.

Using the sex marker developed by Wang et al. [19] and modified by Peñailillo et al. [25], the contemporary plants from the native range (Vietnam, China and Taiwan), and from Japan and Hawaii present both sexes. All 277 contemporary samples from Remote Oceania were identified as female plants, with the exception of 36 plants from Hawaii that were identified as male plants. Hawaii is the only archipelago in Remote Oceania where both contemporary male and female plants are present. In addition, 45 herbarium samples from Remote Oceania were also female. Of the 37 herbarium samples from Hawaii, only a subset of 13 amplified for this marker and were genotyped as female specimens. As indicated in the Materials and Methods section, primers designed to amplify a smaller region of the sex marker yielded results for an additional group of eight herbarium samples that were also genotyped as female plants. It was not possible to determine the sex of 16 Hawaiian herbarium samples. Two herbarium samples (described in [27]), one from the Marquesas Islands and one from the Austral Islands (Rapa), were typed as male plants. Sex marker and ITS-1 results are summarized in Table 1.

**Table 1:** ITS-1 polymorphisms and sex of *B*. *papyrifera* samples.

	Locality	Number of Samples		ITS (polymorphism 99)		ITS (polymorphism 203)						Sex Marker				
		Leaf	Herbarium	Number of leaf samples		Number of leaf samples		Number of herbarium samples				Number of leaf samples		Number of herbarium samples		
				T	C	G	T	G	T	D. S.	N. R.	Female	Male	Female	Male	N.A.
**Asia**	China	4	1	2	2	4	-	-	-	-	1	2	2	-	-	1
	Japan	5	-	-	5	5	-	-	-	-	-	3	2	-	-	-
	Taiwan	19	-	-	19	19	-	-	-	-	-	8	11	-	-	-
	Vietnam	5	-	3	2	5	-	-	-	-	-	2	3	-	-	-
**Near Oceania**	Solomon Is.	-	3	-	-	-	-	3	-	-	-	-	-	3(1)*	-	-
	New Guinea	-	2	-	-	-	-	1	-	1	-	-	-	2	-	-
**Remote Oceania: West**	Fiji	74	4	-	74	-	74	-	4	-	-	74	-	4(1)*	-	-
	Tonga	51	1	-	51	-	51	-	1	-	-	51	-	-	-	1
	Futuna	-	1	-	-	-	-	-	1	-	-	-	-	1	-	-
	Samoa	17	2	-	17	-	17	-	2	-	-	17	-	2	-	-
	Wallis	13	-	-	13	-	13	-	-	-	-	13	-	-	-	-
	New Caledonia	3	-	-	3	-	3	-	-	-	-	3	-	-	-	-
**Remote Oceania: East**	Marquesas	14	2	-	14	-	14	-	2	-	-	14	-	1	1	-
	Cook Is.	-	1	-	-	-	-	-	1	-	-	-	-	1	-	-
	Niue	-	2	-	-	-	-	-	2	-	-	-	-	1	-	1
	Austral Is.	2	7	-	2	-	2	-	7	-	-	2	-	6	1	-
	Pitcairn	2	3	-	2	-	2	-	3	-	-	2	-	3	-	-
	Tahiti	6	-	-	6	-	6	-	-	-	-	6	-	-	-	-
	Rapa Nui	60	5	-	60	-	60	-	5	-	-	60	-	5	-	-
	Hawaii	37	30	18	19	18	19	3	3	22	2	19	18	14(6*)	-	16
	New Zealand	-	2	-	-	-	-	2	-	-	-	-	-	-	2	-
**Other**	Santiago	1	1	-	1	1	-	1	-	-	-	1	-	1	-	-
	**Total**	**313**	**67**	**23**	**290**	**52**	**261**	**10**	**31**	**23**	**3**	**277**	**36**	**45**	**3**	**19**

D.S.: Double signal at indicated position; N.R.: Non-readable sequence; N.A.: No amplification; ()*: sex-typed samples using modified sex marker protocol.

The 57 herbarium and 35 contemporary samples from Oceania were selected for analysis with the *ndhF-rpl32* cpDNA marker. Haplotype cp17 was found in 84 samples (from New Guinea, Fiji, Tonga, Futuna, Samoa, Wallis, New Caledonia, the Marquesas, Cook Islands, Niue, Rapa, Pitcairn, Tahiti, Rapa Nui and Hawaii), in concordance with previously described results [26]. Haplotype cp41 was detected in three male contemporary Hawaiian samples. The scrutiny of a greater number of contemporary samples from Remote Oceania compared to those analyzed by Chang et al. [26], enabled us to detect two new cpDNA haplotypes in ERO derived from the cp17 haplotype from Southern Taiwan. These two new haplotypes were found in two samples: cp49 was detected in one sample from Rapa Nui and haplotype cp50 was identified in one sample from the Marquesas, confirming a direct genetic connection with southern Taiwan (S4 Table).

#### Genetic analysis of contemporary and herbarium samples with microsatellite markers

All 380 samples (313 contemporary and 67 herbarium samples) were successfully analyzed using nine SSR markers. The SSR marker Bro 07 produced erratic amplification with herbarium samples and was therefore excluded from the population structure analyses. In spite of the vegetative propagation of this species in Oceania, a total of 104 different genotypes were identified in the 380 samples based on the nine SSR markers. In the 313 contemporary samples 65 genotypes were identified, while 31 genotypes were identified in 67 herbarium samples. We found eight shared genotypes between contemporary and herbarium samples. Within 339 samples from Remote Oceania, a total of 64 genotypes and 32 private alleles were found. Each SSR presented between one and 16 private alleles (S5 Table). In this region, the 279 contemporary samples exhibited 40 different genotypes, while the 60 herbarium samples collected in the same area presented 32 genotypes (see Table 2). For details about allele frequencies *per* SSR marker and populations see S6 and S7 Tables.

**Table 2:** Number of genotypes in contemporary and herbarium samples of *B*. *papyrifera*.

Geographic Area	Region (country or island)	Genotypes found exclusively in:				Number of exclusive genotypes of the location	Number of genotypes shared with other locations
		Contemporary (C)	Herbarium (H)	Shared (C/H)	Total		
**Asia**	China	4	1	-	**5**	**5**	-
	Japan	5	-	-	**5**	**5**	-
	Taiwan	18	-	-	**18**	**18**	-
	Vietnam	5	-	-	**5**	**5**	-
**Near Oceania**	New Guinea	-	2	-	**2**	**2**	-
	Solomon Islands	-	3	-	**3**	**3**	-
**West Remote Oceania (WRO)**	Fiji	6	4	-	**10**	**8**	**2** (G048; G049)
	Samoa	5	1	-	**6**	**5**	**1** (G058)
	Tonga	7	-	1	**8**	**3**	**5** (G048; G049; G063; G064; G084)
	Futuna	-	1	-	**1**	**-**	**1** (G049)
	Wallis	1	-	-	**1**	**-**	**1** (G058)
	New Caledonia	2	-	-	**2**	**2**	-
**East Remote Oceania (ERO)**	Austral Islands	1	2	1	**4**	**3**	**1** (G063)
	Cook Islands	-	1	-	**1**	**1**	-
	Marquesas	4	1	1	**6**	**3**	**3** (G063; G069; G070)
	Niue	-	1	-	**1**	**-**	**1** (G071)
	Pitcairn	-	-	1	**1**	**-**	**1** (G048)
	Tahiti	4	-	-	**4**	**2**	**2** (G069; G071)
	Rapa Nui	4	-	2	**6**	**5**	**1** (G070)
	Hawaii F	6	8	1	**15**	**10**	**5** (G063; G064; G084; G090; G098)
	Hawaii M	5	-	-	**5**	**5**	-
	Hawaii (non-sexed)	-	6	1	**7**	**4**	**3** (G063; G090; G098)
	New Zealand	-	2	-	**2**	**2**	-
**Other**	Chile	1	1	-	**2**	**2**	-
					**Total**	**93**	**11** (G048; G049; G058; G063; G064; G069; G070; G071; G084; G090; G098)

**Fig 1:**
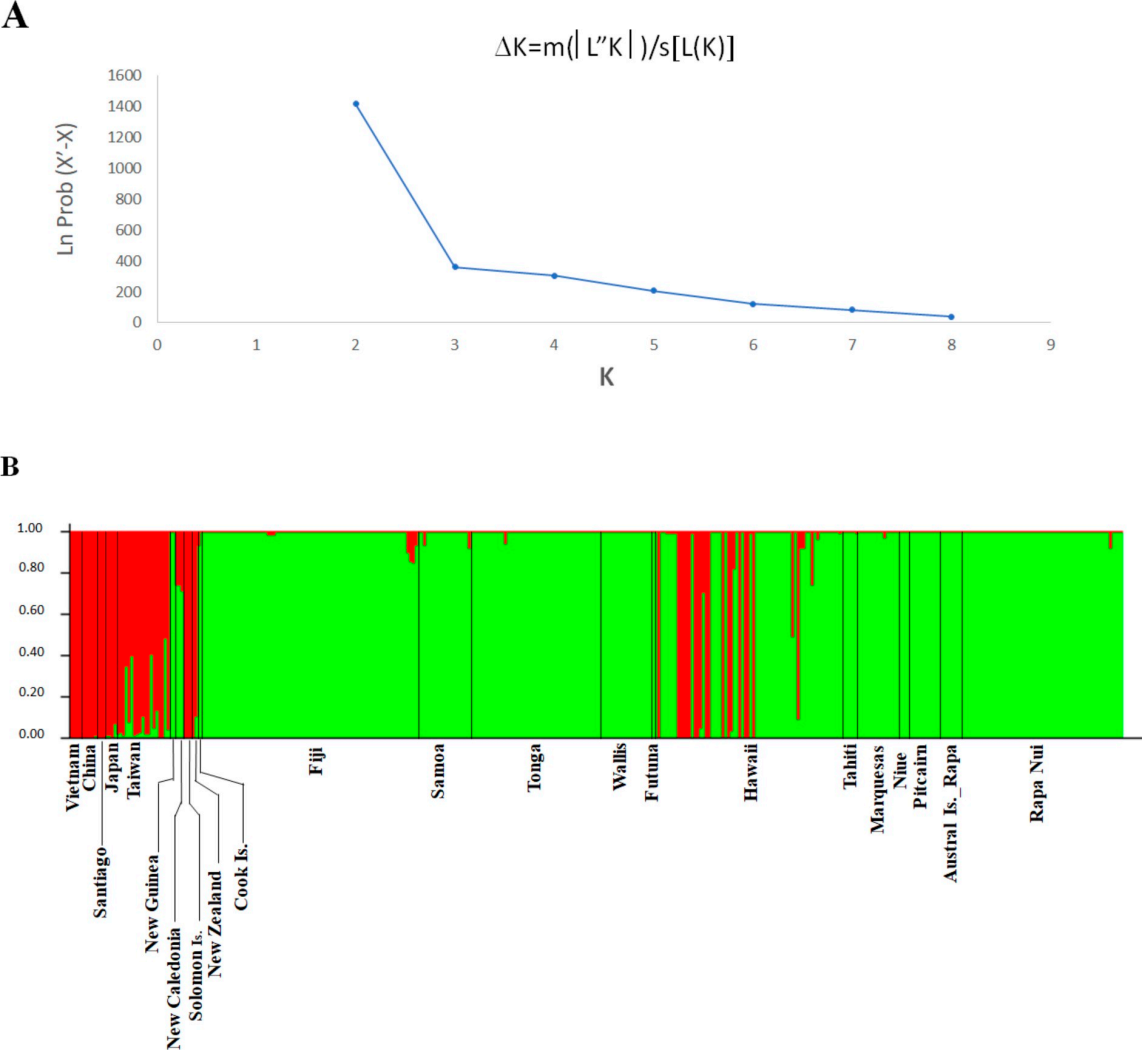
STRUCTURE plot of *B*. *papyrifera* populations. A) Delta K values calculated by Evanno’s method detecting K = 2, as the most probable number of biologically meaningful genetic clusters within the 9-loci data. B) Probabilities of membership in clusters K1 and K2 implemented in Structure. Each individual is represented as a vertical bar, with colors corresponding to membership probabilities in clusters K1 (red) indicating Asian origin, and K2 (green) indicating Pacific genotypes.

The Bayesian analysis of genetic populations indicates two genetic clusters (Fig 1A). All samples from the native range group in one cluster and the samples from the introduced range are grouped in a second cluster, as shown in Fig 1B. The first cluster also contains specimens from New Zealand, the Solomon Islands and Santiago (Chile), all samples reported by their collectors as modern introductions from Asia or indirectly *via* Europe. Some of the Taiwanese samples that group in the first cluster exhibit genetic properties of the second cluster. In contrast, within the introduced range, the sole island group that presents samples of both clusters is Hawaii. However, in these samples no significant mix of clusters is observed within any individual, as observed in samples from Taiwan. In particular, specimens that group in the first cluster correspond exclusively to male individuals, while all those grouping in the second cluster are female samples, revealing a different genetic structure within the *B*. *papyrifera* population of Hawaii, as opposed to the plants from all other Pacific islands.

**Fig 2:**
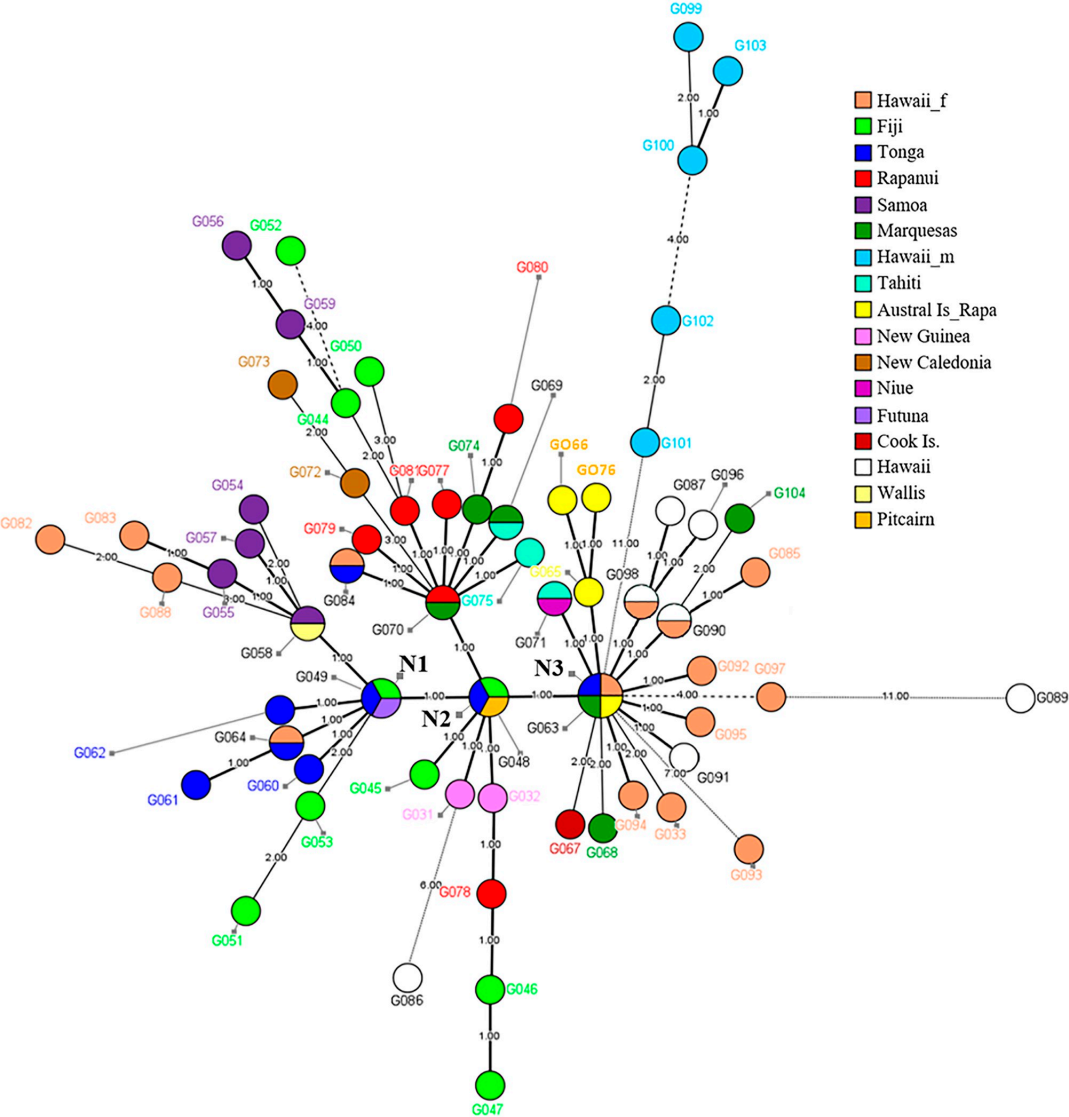
Genetic structure of Pacific *B*. *papyrifera* samples based on Bionumerics analysis. Thickness of lines indicates the degree of closeness between genotypes.

A Minimum Spanning Tree (MST) analysis revealed a complex structure among the 64 genotypes found in Near and Remote Oceania and a close relationship between three central nodes (N1, N2 and N3), each connected through one mutation (Fig 2).

The first node (N1) corresponds to genotype G049 (for details about genotypes, see S7 Table) and is found in Fiji (68 contemporary samples), Tonga (36 contemporary samples) and Futuna (one herbarium sample). Thirteen genotypes diverge from this node, by one to five mutations. Of these, nine genotypes are found exclusively in WRO (Fiji, Tonga, Wallis and Samoa), one is shared by herbarium samples from Tonga and both contemporary and herbarium samples from Hawaii (genotype G064), and three are found only in contemporary samples from Hawaii.

The second node (N2) corresponds to genotype G048 and is found in Fiji (one contemporary sample), Pitcairn (three herbarium and two contemporary samples) and Tonga (one contemporary sample). From N2 a total of 23 genotypes are divergent by one to eight mutations. Of these, two genotypes are found in Near Oceania (New Guinea) and ten in WRO (New Caledonia, Fiji and Samoa). Ten genotypes were found in contemporary specimens from ERO (Rapa Nui, Marquesas Islands and Tahiti), and one genotype (G084) is found in contemporary samples from Tonga and Hawaii.

The third node (N3) is genotype G063, found in Tonga (one contemporary sample), Rapa (one herbarium sample), Hawaii (12 herbarium and eight contemporary samples) and the Marquesas (nine herbarium and one contemporary samples). From N3, a total of 25 genotypes found only in ERO (Marquesas, Cook Islands, Rapa and Hawaii), are derived. N3 presents a complex pattern where 16 genotypes are separated from the central node by only one or two mutations. One of these genotypes is found in Tahiti (four herbarium samples) and Niue (two herbarium samples). Nine genotypes are divergent by four to 15 mutations from the N3 node. This node includes 18 Hawaiian genotypes, where female, male and non-sexed samples are shown in different colors. All five male genotypes from Hawaii (light blue) group on a single branch, separated by 11 to 15 mutations from this central genotype. Most of the non-sexed samples share the same haplotype as or are closely related to female samples. However, one of the Hawaiian samples that could not be sexed differs by at least 11 mutations from the closest female sample. Interestingly, branching from the N3 node, we identified genotype G104, which corresponds to the male plant from the Marquesas. The relationship between the male Hawaiian plants and Asian samples is shown in S1 Fig.

**Fig 3:**
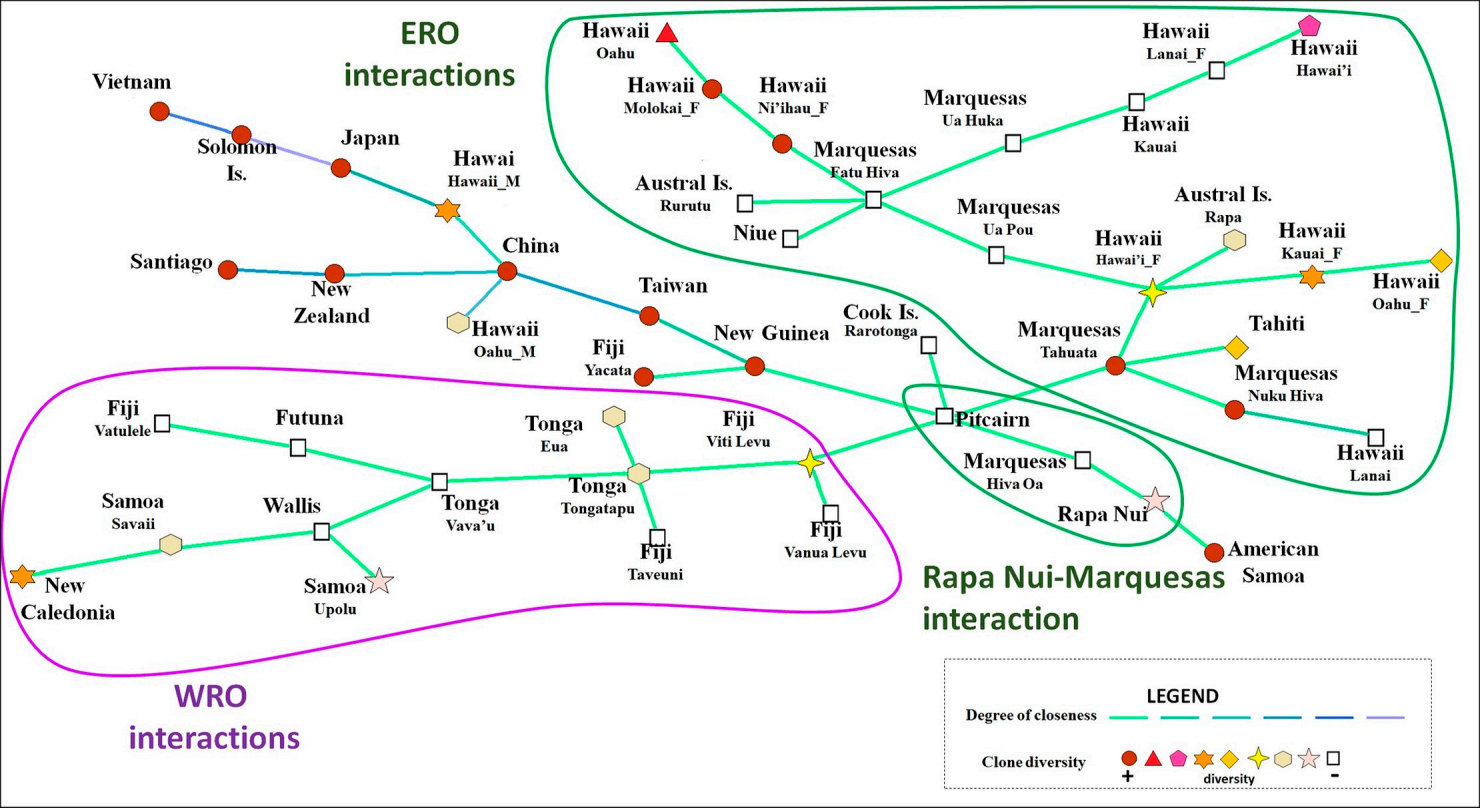
Genetic network and geographic connections between paper mulberry genotypes. Network analysis based on Goldstein distance. Minimum Spanning Tree based on pairwise genetic distances ((δμ)2) arranged approximately by geographic layout of sampling locations. The degree of ‘connectedness’ between localities or islands is indicated by the color of the connecting lines. Lighter colors and decreasing thickness of edges indicate decreasing connectedness. The shape of nodes is indicative of clonal diversity. Diversity is expressed as the ratio between the number of samples and the number of genotypes for each locality. Color and shape of nodes indicate clone diversity in each locality, where the red circle indicates maximum diversity (+) and the white square lowest diversity (-). The branch comprising all localities in WRO is encircled by a purple line and branches in ERO are circled with a green line.

Analysis using EDENetwork (Ecological and Evolutionary Networks) software based on the Goldstein distance resulted in a network where nodes representing islands or “localities” are linked based on genetic data and geographic coordinates (Fig 3). The analysis shows the genetic relationships between the localities of the native and introduced range. One branch groups all samples from the native range or samples known to derive from mainland Asia, while all other branches connect localities within the introduced range. There is a high degree of closeness or relatedness between samples within Asia and with samples known to have been introduced from the native range in recent times (New Zealand, Solomon Islands and Santiago, Chile). The analyzed samples from the native range do not reflect the genetic diversity of this large region, as our sampling is only referential. There is an intermediate genetic closeness between the samples from Taiwan and New Guinea, representing the translocation of paper mulberry plants “Out of Taiwan” and into Near Oceania. In the introduced range, a high degree of closeness between all samples is observed as indicated by the green lines. The only exception are contemporary male plants from Hawaii, represented by two branches within the Asian sample set, one including accessions from the island of Hawai’i, and the other from Oahu. Both are derived from the China node, but present different degrees of closeness (Fig 3).

In the resulting network we observe that the maximum degree of connectivity (*k*) is *k* = 4, with China identified as a central node linking directly to Taiwan and indirectly to Vietnam, all in the native range. The latter branch also includes plants from outside the native range that represent historic (Japan) and modern introductions, corresponding to plants from the Solomon Islands and the male samples from Hawaii. On a separate branch we find samples from New Zealand and Santiago (Chile), which also represent introductions within the last 150 years.

Samples from Taiwan are the sole link between the native and introduced range in the Pacific. Taiwan is connected directly to samples from New Guinea, in Near Oceania. New Guinea shows two connections (*k* = 3) that establish a link to Remote Oceania. One of these links is to a herbarium sample from Fiji (Yacata Island) in West Polynesia and the other link connects New Guinea through Pitcairn to all other samples in East Polynesia. The maximum degree of connectivity found in the introduced range is *k* = 5 present in Pitcairn and in Fatu Hiva (Marquesas Islands). Samples from Pitcairn are connected directly with samples from New Guinea, Fiji, the Cook Islands and two islands from the Marquesas. Samples from Fatu Hiva connect a subgroup of ERO islands, including other islands from the Marquesas, plants from most of the Hawaiian Islands, the Austral Islands and Niue.

Within the introduced range of Near and Remote Oceania we find three localities with high connectivity (*k* = 4). These are Tongatapu (Tonga), the island of Hawai’i (female samples) and Tahuata in the Marquesas. Four other localities present connectivity of *k* = 3, corresponding to New Guinea, Wallis, Vava’u in Tonga and Viti Levu in Fiji. The branches grouping localities from WRO have less clonal diversity than branches with localities from ERO, as seen by node shape and the number of branches of the network (Fig 3).

All samples from WRO are grouped on a single branch that connects Pitcairn to Viti Levu (Fiji) and through several steps with other islands in Fiji (Taveuni and Vatulele), Tonga (Vava’u and ‘Eua), Futuna, Wallis, Western Samoa (Savai’i and Upolu) and New Caledonia. Samples from Savai’i and Upolu islands are derived from those from Wallis. New Caledonia plants derive from Savai’i.

The three other branches from Pitcairn connect to all genotypes from ERO and two genotypes from WRO, found in American Samoa and Niue. One of these branches connects Pitcairn with the Cook Islands. The second and third branches connect Pitcairn with two islands in the Marquesas. One of these branches links Pitcairn to Hiva Oa and to derived genotypes from Rapa Nui and American Samoa. The other branch that links Pitcairn to the Marquesas via Tahuata Island (*k* = 4) includes several derived genotypes that are associated to other Marquesan islands, to Tahiti, to several islands in Hawaii, to Rapa and Rurutu in the Austral Islands and to Niue.

The greatest clonal diversity is observed in two islands of the Marquesas (Tahuata and Nuku Hiva), three islands from the Hawaiian archipelago (Ni’ihau, Molokai and Oahu) and in American Samoa, located in WRO. However, the diversity of the node from American Samoa is misleading since this island is represented by a single sample, and therefore node shape is not representative of greater clonal diversity. One sub-branch from Tahuata connects with the island of Nuku Hiva from the same archipelago and to a derived genotype from non-sexed samples from Lana’i (Hawaii). The second sub-branch links Tahuata to Tahiti. The third sub-branch connects to the female samples from the island of Hawai’i and presents *k* = 4, connecting to Rapa in the Austral Islands, and on a separate sub-branch, to female samples from Kauai and Oahu in Hawaii. Additionally, the Hawai’i node joins via Ua Pou in the Marquesas (*k* = 4) to a *k* = 5 node in Fatu Hiva. Fatu Hiva links to islands in Hawaii, Niue, Rurutu (Austral Islands) and the Marquesas. One sub-branch from Fatu Hiva connects to Niue. A second sub-branch connects to Rurutu. The third sub-branch connects to Ni’ihau and derived genotypes of female samples from Molokai and non-sexed specimens from Oahu, all from Hawaii. The fourth sub-branch links Fatu Hiva to derived female samples from Ua Huka and also three genotypes in Hawaii (non-sexed samples from Kauai, female samples from Lanai and non-sexed samples from Hawai’i) (Fig 3).

## Discussion

In this work, we present the analysis of *B*. *papyrifera* specimens with the aim of understanding its dispersal history in Remote Oceania and inferring the associated human movements. Paper mulberry is unique among the commensal species studied, because it is not primarily a food item, but a source of fiber for making barkcloth textiles which are of great cultural importance [20]. This paper integrates data obtained from the analysis of 380 contemporary and herbarium samples using four kinds of genetic markers. All of these are simple markers, and each provides a different and complementary perspective that is relevant for the reconstruction of the dispersal history of this species in the Pacific. Integration of data provided by the chloroplast and nuclear (ITS-1, sex and microsatellite) markers have shown robust and consistent results.

The analysis of the samples with the sex marker confirmed that contemporary plants found today from New Guinea to Rapa Nui are all female plants, with the exception of those from Hawaii, where male plants are also present. Male plants in Hawaii share haplotypes with the Asian mainland and Japan, suggesting an independent introduction to this archipelago, probably during historic times [24]. Among the herbarium samples, two specimens from the Remote Oceanic cp17 haplotype are male (samples BISH161297 from Rapa and BISH161281 from the Marquesas), as discussed in Payacán et al. [27]. Results obtained with the three nuclear and chloroplast markers are consistent in showing a general homogeneity of paper mulberry within the Pacific at large, in clear contrast with the diversity found within the native range. This homogeneity is a reflection of a single origin and dispersal from the Taiwanese homeland, congruent with the linguistic and archaeological evidence [43].

New sequencing technologies such as Next Generation Sequencing (NGS) have been used to study the dispersal patterns of other species through Remote Oceania, such as rats [44] and dogs [45, 46], which are well studied animals whose reference genomes have already been published. Until recently, for NGS analyses, the use of a reference genome was essential and NGS applied to herbarium samples is currently under development [47, 48, 49] and focused mainly on well-studied plant species. To date chloroplast DNA genomic sequences of *B*. *papyrifera* (NCBI Reference Sequence: NC_035569.1) are available, but not so a nuclear reference genome. The closest genome published is from *Morus notabilis* (2n = 14) [50], a Moraceae species with a relatively small genome (320 Mb) compared to *B*. *papyrifera* (2n = 26, 503 Mb) [51] or a 2C value of 702 pg compared to 1022 pg, respectively [52]. The use of microsatellites is appropriate and has advantages for our study because of the higher mutation rate of microsatellite motives compared to the mutation rate of single nucleotide polymorphisms [53] that are evaluated in high throughput techniques.

Using microsatellites with a standard PCR approach, genetic diversity within Remote Oceania was revealed, despite the asexual propagation of paper mulberry and the short time scale since its human-mediated introduction into the Pacific (less than 10,000 years). This genetic diversity is evidenced by two kinds of independent analyses, i.e. network analysis based on Bionumerics and Goldstein distance. The first analysis highlights the internal structure of genotypes from paper mulberry populations within Remote Oceania revealing three main dispersal hubs, while the latter analysis shows the degree of genetic closeness of plants from each sampled geographic locality and their connections with the Asian homeland.

The inclusion of a large number of contemporary accessions and additional herbarium samples to the ones published in Payacán et al. [27], shows a more complex relationship of the detected genotypes (Fig 2). Previously, in Payacán et al., [27] we observed three main genetic groups (GG) among herbarium samples from Asia and Oceania. One group comprised the native range and two groups included samples from Remote Oceania. One of these latter groups (GG2) included samples from Samoa, Tonga and Futuna and the third group (GG3) included samples from Pitcairn, Niue, New Guinea, the Cook Islands, Marquesas, Austral Islands and Rapa Nui. The link between these two groups was Pitcairn. In this study we again find that Node 1 (N1) includes the same localities from GG2 (from WRO) but incorporates the Fijian islands in a central position, displacing samples from Samoa to a derived position from this central node. In contrast, the enlarged sample set for ERO now splits GG3 from the herbarium study into two nodes (N2 and N3). N2, represented by genotype G048, includes the samples found on Pitcairn that again links WRO with ERO. By including additional contemporary samples from new localities, this node now also comprises Fiji and Tonga, reinforcing the central position of Pitcairn in articulating WRO and ERO. Genotype G048, linking to genotypes found on relatively isolated islands in ERO, Near Oceania and WRO, suggests dispersal of a limited number of plants into Remote Oceania. The general diversification pattern that includes Tonga on the three nodes and repeatedly links localities of WRO to those in ERO, like New Guinea to Rapa Nui, New Caledonia to Marquesas and Tonga to Marquesas and Hawaii, supports the notion that isolated localities such as some islands in the Marquesas and Rapa Nui may have maintained genotypes that have been lost in WRO.

The derived genotypes of N2 include the same regions or island groups that were linked in the former study of herbarium specimens, such as New Guinea and Rapa Nui. Genotype G070, shared by two samples from Tahuata in the Marquesas and several samples from Rapa Nui, is derived by one mutation from the central N2, and gives rise to several genotypes connected to Fiji and Samoa and shared with the Marquesas. This suggests that the islands Tahuata and Rapa Nui have maintained ancestral Pacific genotypes until today. Some of these derived genotypes are also found in New Caledonia, Samoa and Fiji, suggesting that G070 is an ancient genotype. The presence of samples from Rapa Nui in this node may reflect the relatively early arrival or translocation of paper mulberry plants to this island, in spite of its geographic location on the southeastern margins of the Polynesian triangle.

Node 3 (genotype G063) includes genotypes from Tonga and localities from ERO. Tonga is the only island group that is represented in the three nodes, reflecting its importance as a dispersal center or interaction hub between West and East Remote Oceania. The derived genotypes from N3 show a large genetic diversity of female plants within the Hawaiian archipelago, and a connection between the genotypes found on the Austral and Cook Islands. In this node one genotype links a group of plants collected at the Musée de Tahiti et des Îles which share a common genotype with plants from Niue (herbarium sample). The most distant samples (by at least 11 mutations) in this node cluster all male specimens and one non-sexed sample from Hawai’i, indicating again that their genetic signature reflects a different history (S2 Fig). This node groups most islands from Central Polynesia and Hawaii. The absence of both contemporary and herbarium accessions from the Gambier Islands precludes an analysis of the relationship between plants from Pitcairn and the Gambier Islands, the Gambier Islands and Rapa Nui, or the Marquesas and the Gambier Islands.

### Loss of diversity over time

Our results show a loss of genetic diversity in contemporary plants when herbarium and contemporary samples from Remote Oceania are compared. Integration of results from herbarium and contemporary samples was possible with nine microsatellites. One marker (Bro 07) was excluded because it did not amplify DNA from herbarium samples consistently. The analysis of the collection with nine microsatellites supports diversification of clones within Remote Oceania. Our data show that contemporary paper mulberry plant samples are less diverse than herbarium samples, indicative of a loss of genetic diversity over time. Our relatively large collection of 279 contemporary samples from Remote Oceania exhibits 40 genotypes, while the smaller collection of 60 Remote Oceania herbarium samples exhibits 32 genotypes. The ratio between the number of contemporary samples to genotypes is seven to one, while this ratio for herbarium samples is close to two to one (1.9), meaning that in relative terms the smaller herbarium collection exhibits higher genetic diversity. It is difficult to assess the reasons for this loss of diversity, which may be explained by the recent decline of tapa culture during the twentieth century on some islands, replacement of plants on others, local disappearances and, finally, incomplete sampling on some of the most remote islands.

### From Taiwan to Remote Oceania

**Fig 4:**
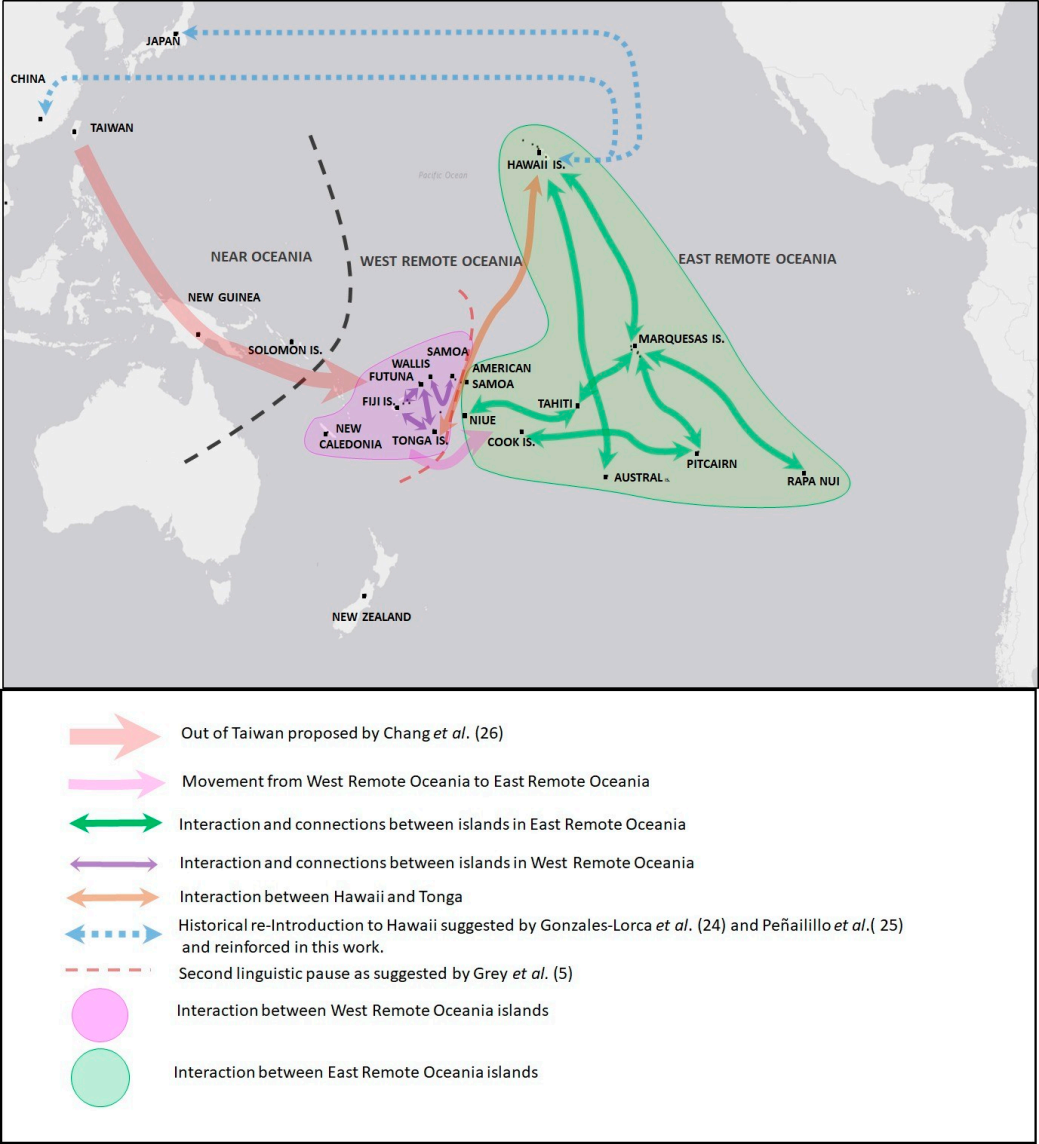
Map of the Pacific showing prehistoric and historical dispersal of paper mulberry in Oceania. Integrated results of the dispersal routes as postulated by Chang et al. [26], Payacán et al. [27] and this work.

For the first time we detect genetic diversity and genetic structuring among contemporary plants within Remote Oceania, which allow us to establish connections between island groups, indicating translocations and suggesting migration routes (Fig 4). We illustrate the prehistoric and historical dispersal of paper mulberry in Oceania as derived from previously published data [26, 27] and this work. The Structure analysis and the MST network that link genetic distance with geographic locations are consistent with the information provided by the cpDNA showing that the Pacific samples share genotypes with Taiwan as the nearest genetic population within Asia [26]. Both analyses highlight the general homogenous genetic background of the Remote Oceanian populations, with the exception of the male Hawaiian plants. In the MST network we observe that Taiwan is directly connected to Near Oceania through New Guinea, as evidenced by a female herbarium specimen collected in 1937 that presents the cp17 haplotype. New Guinea is directly connected to Remote Oceania through Pitcairn. This relationship between samples from New Guinea and Pitcairn represents the survival of old genotypes on Pitcairn Island due to centuries of isolation after initial colonization by Austronesian speaking peoples. We suggest that these genotypes were probably lost on other islands that represent the intermediate steps of dispersal and migration. We do not propose a direct migration route from New Guinea to Pitcairn. A similar situation can be inferred for the connection observed between samples from New Guinea and accessions from the island of Yacata in Fiji. This also seems to be a case of survival of old genotypes on relatively isolated islands. Using the cpDNA *ndhF-rpl32* region we found two new haplotypes (cp49 and cp50) which derive from the cp17 haplotype found in all other analyzed samples (S4 Table). Our large contemporary and herbarium collection from Remote Oceania does not present any other haplotypes. Haplotype cp34, detected by Chang et al. [26] in New Guinea and linked to haplotypes from Indochina, is conspicuously lacking in our data from Remote Oceania. The absence of the cp34 haplotype in Remote Oceania suggests that the introduction of this plant variant was an independent event limited to Near Oceania, and was not part of the dispersal further east into Remote Oceania, possibly because of different timing or different peoples involved in its transport. The sole presence of genotypes derived from cp17, native to Taiwan, confirms that paper mulberry plants in Remote Oceania originate exclusively from Taiwan. Our limited number of samples from Taiwan and Near Oceania precludes any deeper analysis on the relationship between New Guinea and the homeland in Taiwan and between New Guinea and Remote Oceania.

Analyzing the relationships within Remote Oceania, we observe a clear west to east structuring (Figs 2 and 3) that is consistent with archaeological, linguistic and genetic data in terms of the settlement direction of this large geographic region [43]. We suggest that the genetic structure observed in these paper mulberry accessions represents one-way movements associated with settlement or colonizing events in the past and not interaction or exchange networks, because the plant itself does not have ritual value. Crops important for survival and cultural reproduction were probably aboard all colonizing canoes [43], although probably not part of later inter-archipelago commercial networks or part of ritual exchanges of high valued objects, such as textiles, adzes, whale teeth, shells and other items between established settlements. We propose, that it is exclusively the final product (i.e. barkcloth textiles) that embodies high cultural value and therefore could have been widely exchanged among islands in the past as part of the extensive interaction networks.

Contrary to our expectations, we did not observe a gradual decrease of genetic diversity from West to East Remote Oceania. Of the 64 genotypes found in Remote Oceania, 20 genotypes are exclusive to WRO; 40 are exclusive to ERO, and four genotypes are shared by WRO and ERO. This might be explained as the result of a rapid translocation of the plants and the people that carried them from WRO to ERO, in agreement with the genetic closeness shown in Fig 3, revealed by the connections between geographic locations summarized in S3 Fig, and is consistent with archaeological studies that suggest a short and recent chronology for the settlement of East Polynesia (e.g. [4, 54]).

Tonga is the only island group represented in the three nodes, reflecting its importance as a dispersal or interaction hub between West and East Remote Oceania. Our data is suggestive of Tonga being the main dispersal hub of paper mulberry into ERO. We found a general diversification pattern that includes Tonga on the three nodes and repeatedly links localities of WRO to those in ERO, such as New Guinea to Rapa Nui, New Caledonia to Marquesas and Tonga to Marquesas and Hawaii. This result suggests that relatively isolated localities such as some islands in the Marquesas and Rapa Nui may have maintained ancient populations /genotypes that have been lost in WRO. In this work and in Payacán et al., [27], we found Pitcairn plants in a pivotal position between WRO and ERO. In addition, Pitcairn accessions linked with genotypes from New Guinea in Near Oceania. Targeting islands of Central Polynesia, which are under-represented in our sample set, should permit us to identify the intermediate “stepping stones” and increase the resolution of the connections between WRO and ERO.

### West Remote Oceania

Within Remote Oceania major hubs or nodes are apparent, showing complex interaction networks among islands (Fig 4 and S3 Fig). One of these hubs includes Fiji and Tonga, consistent with the existence of an Ancestral Polynesian Culture (APC) or homeland [7]. The islands of Fiji and Tonga show a number of related genotypes (Figs 2 and 3). In addition to being part of the three central dispersion nodes (Fig 2), they share the largest number of genotypes. This might reflect ancient connections during early settlement of WRO or the expansion and conquest of Fijian islands by the formerly powerful “Tongan Maritime Empire” that extended its influence to the west to Fiji, Wallis and Futuna and to the east as far as Central Polynesia [55]. In a study of the distribution of volcanic glasses Burley et al. [56] suggest the existence of an extensive network and inter-island relationships between Tonga and the Lau island in Fiji in at least the initial phases of ancestral Polynesian development, in concordance with linguistic data [57].

It is of note that Samoa does not share any genotypes with either Fiji or Tonga. Our data support the proposed divergent histories of Samoa and Tonga [58]. In concordance with our data, several authors observe significant differences between Tongan and Samoan material culture during the early settlement phase, such as ceramics, adzes, use of shells and volcanic glass [58, 59]. For example, Burley et al. [56] also highlight the absence of Samoan volcanic glass in Tongan archaeological sites, as well as the reverse. According to these authors, the volcanic glass distribution strongly implies regional isolation of the two archipelagoes from at least 2500 years into later prehistory [56].

The relationship observed between genotypes from Samoa with those from New Caledonia may represent the survival of ancient genotypes that reflect connection that date back to Lapita colonization voyages, or to back migrations from Western Polynesia. Our samples from New Caledonia are from the Museum of New Caledonia and are derived from plantations from rural localities of Poindimié and Canala on the east coast. As far as we know, they are not related to recent immigrants from either Wallis or Futuna. Surprisingly, the neighboring islands of Futuna and Wallis do not share the same genotypes and are connected solely through Tonga. This may also be the result of the expansion of the Tongan Empire.

### Central and East Remote Oceania

Additional important dispersal hubs are present on the Marquesas archipelago, particularly the southern islands of Fatu Hiva and Tahuata, linking this archipelago to Hawaii and Pitcairn. Radiocarbon dating indicates a rapid (70–265 years) colonization between the Marquesas, Pitcairn, the Society Islands, Hawaii and Rapa Nui [60] with inter island exchange networks lasting until about the 14^th^ century AD [61]. The links between archipelagos such as Hawaii and the Marquesas, and between Hawaii and the Austral Islands are consistent with the archaeological evidence of long-distance trading networks and transport of lithic artifacts among these island groups [62, 63]. Larsen [64], in a phylogenetic reconstruction of barkcloth traditions, suggests close ties between marginal Polynesia, particularly between Rapa Nui and Hawaii, where both are also linked to the Marquesan and Mangarevan traditions. The connections observed in our study through the genetic analysis of paper mulberry plants, also show ties between Rapa Nui and Marquesas and between the Marquesas and Hawaii, although we do not see close ties between Rapa Nui and Hawaii. This may be explained by the fact the Larsen [64] compared cultural features that despite having a common history may develop convergently from a common ancestral tradition and cannot be compared to genetic descent. Our genetic data represent past interactions and survival of related plants; however, convergent developments cannot be inferred.

Genetic data on human dispersal routes within Remote Oceania are scarce, as most human genetic studies have focused on the origins of Pacific peoples from East or Southeast Asia to Near Oceania, rather than on their regional variation or dispersal within Remote Oceania. According to Chapman [65] the neglect of the study of East Polynesian patterns of dispersal maybe partly the result from the acceptance of genetic Polynesian homogeneity as initially established by Hagelberg and Clegg [66] and Sykes et al. [67]. The studies based on morphological differences between East Polynesian populations, particularly those based on craniometric analyses, are more informative [65, 68, 69]. These models suggest that Hawaiian populations have close ties with Marquesan populations, as also formerly suggested by linguistic and biological evidence, while Easter Island was probably settled from the Tuamotus through Mangareva, but not directly from the Marquesas [70]. The Central Polynesian interaction sphere included the Society Islands, Tuamotus and the southern Cook Islands [65].

A recent re-analysis of the genetic relationships and colonization routes inferred from the analysis of Pacific rat (*R*. *exulans*) mitochondrial DNA from archaeological bones suggests an expansion from the Austral Islands to Rapa Nui [42]. Unfortunately, other studies on commensal animal or plant species, such as chickens [71], dogs [45, 46] or taro [72], either do not provide sufficient detail on the genetic diversity within Remote Oceania to infer dispersal routes, or the species did not reach the islands of Marginal Polynesia, such as chickens in New Zealand, or dogs on Rapa Nui. In our data, we find contemporary genotypes from Rapa Nui derived directly from one genotype found on Hiva Oa in the Marquesas (Fig 3). The genotype from American Samoa was found to derive from a genotype from Rapa Nui, suggestive of an ancestral genotype that reflects the expansion from WRO to ERO. We did not find this genotype in intermediate islands in our collection; this observed relationship is consistent with a scenario of dispersal from West Polynesia into the central East Polynesia, and from there to the extremes of the Polynesian triangle, particularly to Rapa Nui and Hawaii [44].

We lack sufficient data from Central Polynesia, mostly from the Cook Islands and the Society Islands to understand the relationship of this region with marginal East Polynesia, specifically Rapa Nui. Samples from Pitcairn Island in our study are, to our understanding, the closest representative genotypes of Central Polynesia. The absence of extant paper mulberry plants and herbarium samples from the Tuamotu and Gambier islands precludes a deeper analysis of the relationships between these islands and Rapa Nui. We also detect numerous interactions among the Marquesas Islands, in agreement with evidence of inter-island exchanges of stone tools documented through geochemical analysis for the late Marquesan society [73].

### Male samples in Hawaii and recent introductions in different locations

Most Hawaiian specimens show either a clear Asian or Pacific genetic make-up and a small minority of specimens present a mixed genetic pattern (Fig 1). This suggests that sexual reproduction is uncommon within Hawaii, despite the presence of male and female plants growing in proximity on the same island. One possible explanation is that male and female plants do not flower at the same time. For example, when sampling for this study (June 2012) only male plants were observed flowering on Oahu and Hawai’i. Other plants that were growing nearby and later sexed as female, were not flowering. The male plants found in Hawaii are genetically heterogeneous, comprising five genotypes found on only two islands in the archipelago. As suggested formerly (24–26), the genetic make-up of the male Hawaiian plants may represent independent introductions from China to Oahu and at least one introduction from either China or Japan to Hawaii by contracted workers during the nineteenth and early twentieth centuries. The presence of Hawaiian genotypes on all three nodes and additionally two shared genotypes between Tonga and Hawaii, may represent several translocations layered in different time periods. The shared genotypes G064 on N1 and G084 on N2, respectively (Fig 2) may represent modern introductions in the late twentieth century.

## Conclusions

Our data, based on the combined analysis of extant and herbarium paper mulberry samples from Oceania, is the result of a comprehensive sampling of 33 islands of Remote Oceania, and compared to samples from New Guinea, the Solomon Islands and Asia. We demonstrate the existence of genetic diversity and genetic structure in paper mulberry in Remote Oceania, despite its vegetative propagation and short timespan since its introduction into the region by prehistoric Austronesian speaking colonists. Additionally, based mainly on microsatellite markers, we detect that extant plant populations have lower genetic diversity than accessions from herbarium collected during the early twentieth century. The observed genetic structure reveals a general west to east trend in the dispersal of the plant, in agreement with known archaeological, linguistic and other genetic data. Our data also detect a complex structure of three central dispersal hubs linking West Remote Oceania with East Remote Oceania. The plants found on the island of Pitcairn, in Southern Marginal Polynesia, due to its long isolation, seem to hold remnants of very ancient genotypes that represent the genetic connection between Near and Remote Oceania. As for the genetic structure within WRO, we observe a close relationship between Tonga and Fiji, while Samoa, Wallis and New Caledonia seem to represent a separate network. Plants from Tonga (Tongatapu) and Fiji represent the genetic link between WRO and ERO. As for the genetic structure within ERO, we distinguish dispersal events that mirror migration and interaction networks as observed from archaeological data, such as the repeated relationship between the Marquesas and Hawaii, Marquesas and Central Polynesia (Tahiti) and with Marginal Southern Polynesia (Marquesas and Hawaii with the Austral Islands, Marquesas with Pitcairn and with Rapa Nui). In this way, the genetic information confirms that the Marquesas archipelago is an important connecting hub within East Remote Oceania, consistent with archaeological and linguistic data. Despite the short time span in which the dispersal of paper mulberry occurred and the clonal nature of its propagation in the Pacific, the genetic study of the dispersal of this fiber plant is a useful proxy for the Austronesian expansion, from its native region (Taiwan) throughout introduced range in the Pacific. The genetic connections detected in contemporary and herbarium samples from paper mulberry reflect prehistoric human movements between multiple islands in Remote Oceania and, to date, provide a more comprehensive picture than other model species. In contrast, textiles made of the bark from this plant may represent evidence of exchange networks of prestige objects, which are unlikely to persist in the archaeological record. Therefore, the genetic analysis of ethnographic textiles from museum collections represents the closest approach to past plant material from this species and could add complementary insight focused on eventual exchange networks.

## Supporting information

S1 Fig Map showing location of islands of provenance of the samples examined in this study. (TIF)

S2 Fig Genetic structure of Asian and male Hawaiian *B. papyrifera* samples (based on Bionumerics analysis). (TIF)

S3 Fig Map showing inter-island connections between localities based on *B. papyrifera* genotypes to infer plant dispersal and human movements in the Pacific. Map constructed based on data from the Minimum Spanning Tree using Goldstein distance. The black dotted line divides Near and Remote Oceania. Lines indicate genotype connections and not directionality. The red line shows connections between the native range and the introduced range. Green lines indicate connections among ERO islands and /or island groups. Purple lines indicate connections among WRO islands and their connection to ERO. The blue dotted lines indicate the connection between Hawaiian Islands and the native range in recent times.
(TIF)

S1 Table Sample codes of contemporary samples, provenance and collection coordinates. (DOCX)

S2 Table Herbarium samples included in this study with codes, field collection number, geographic origin, collectors and year of collection. (DOCX)

S3 Table Sequences and annealing temperature of primers used in this work. (DOCX)

S4 Table cpDNA haplotypes found on different islands and number of samples per haplotype. (DOCX)

S5 Table Private alleles found in Remote Oceania genotypes for each microsatellite marker. (DOCX)

S6 Table Allele frequencies found on different islands in Near and Remote Oceania for each microsatellite. (DOCX)

S7 Table Microsatellite genotypes found on different islands and characteristic alleles for each microsatellite marker. (DOCX)

S1 Text Description of sampling permits and approvals. (DOCX)
