# Supplementary figures and images for: Human mediated translocation of Pacific paper mulberry [Broussonetia papyrifera (L.) L’Hér. ex Vent. (Moraceae)]: Genetic evidence of dispersal routes in Remote Oceania
Source: PLoS One. 2019 Jun 19;14(6):e0217107. doi: 10.1371/journal.pone.0217107 (PMC6583976; doi:10.1371/journal.pone.0217107)

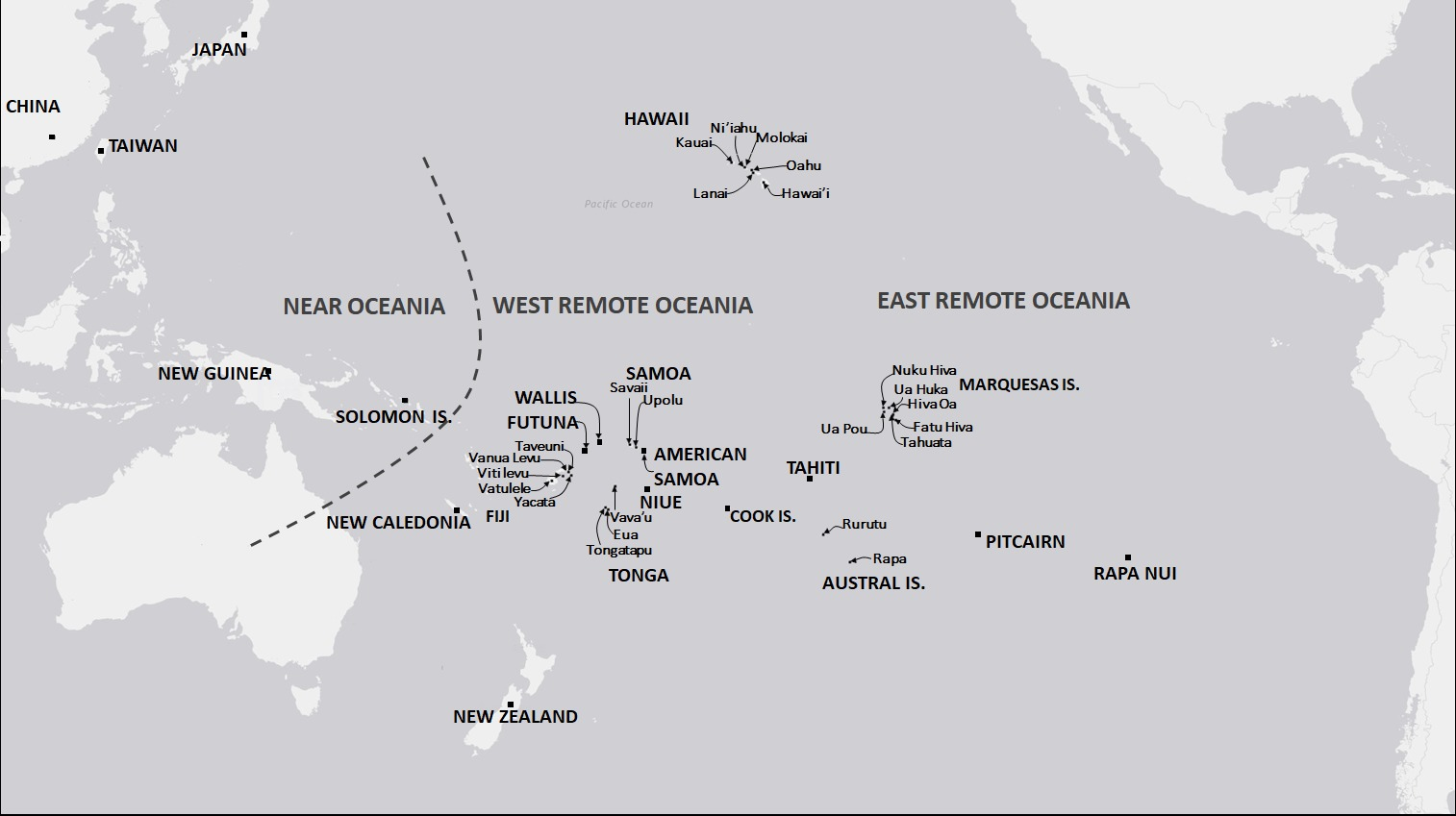

Supplement: S1 Fig — (TIF) [file pone.0217107.s001.tif]

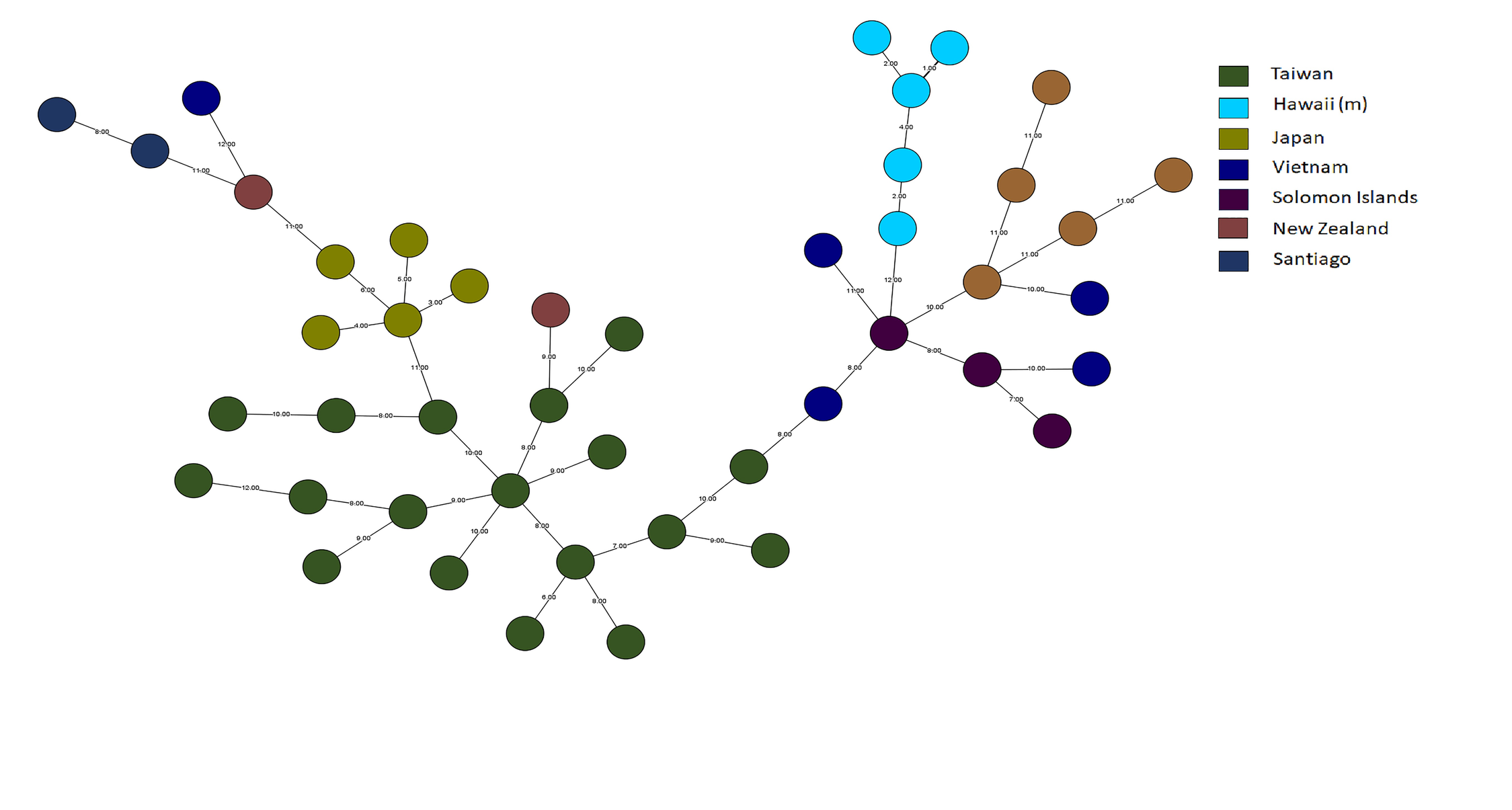

Supplement: S2 Fig — (TIF) [file pone.0217107.s002.tif]

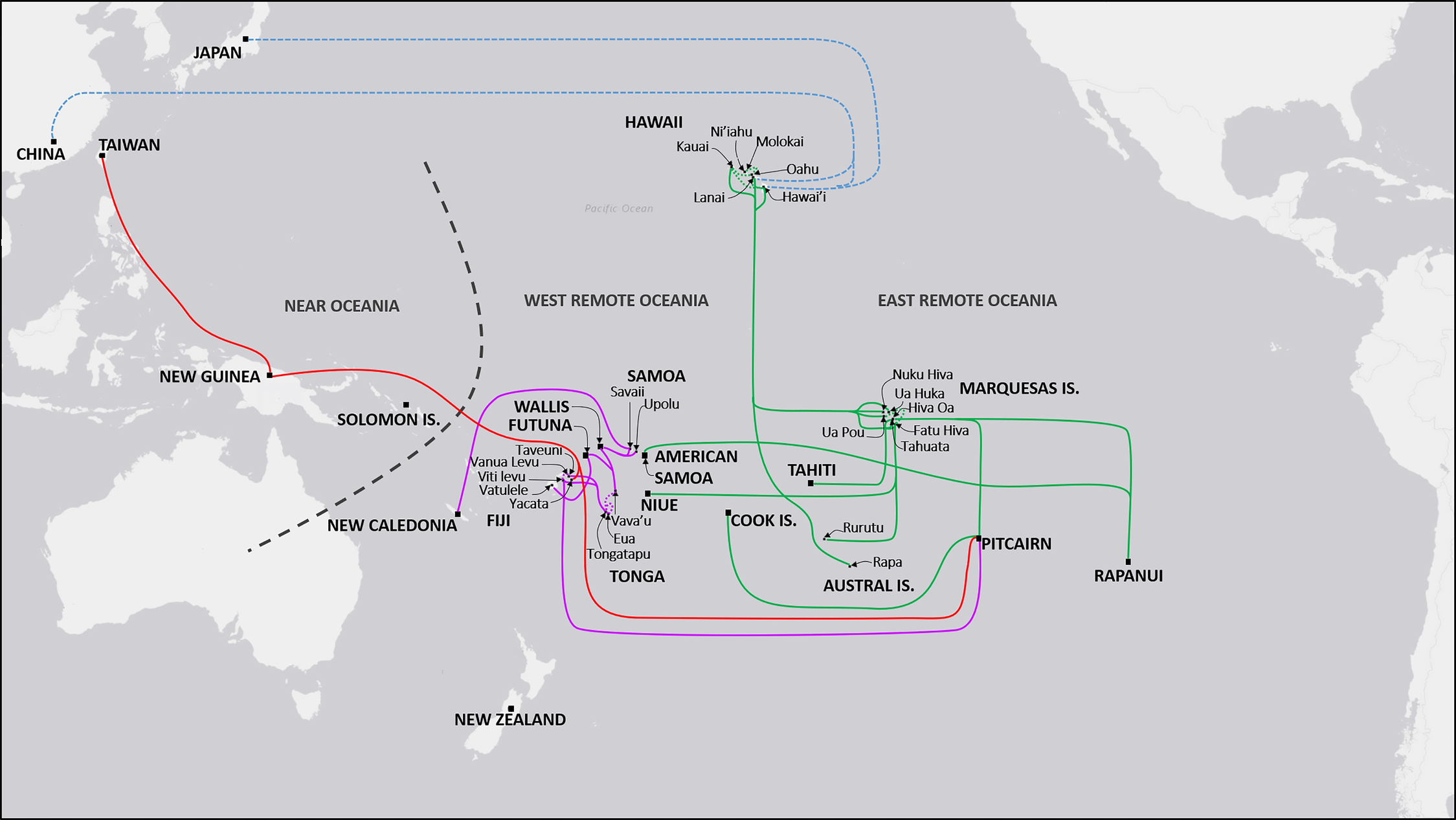

Supplement: S3 Fig — Map constructed based on data from the Minimum Spanning Tree using Goldstein distance. The black dotted line divides Near and Remote Oceania. Lines indicate genotype connections and not directionality. The red line shows connections between the native range and the introduced range. Green lines indicate connections among ERO islands and /or island groups. Purple lines indicate connections among WRO islands and their connection to ERO. The blue dotted lines indicate the connection between Hawaiian Islands and the native range in recent times. (TIF) [file pone.0217107.s003.tif]
