# Supplementary material for: Human mediated translocation of Pacific paper mulberry [Broussonetia papyrifera (L.) L’Hér. ex Vent. (Moraceae)]: Genetic evidence of dispersal routes in Remote Oceania
Source: PLoS One. 2019 Jun 19;14(6):e0217107. doi: 10.1371/journal.pone.0217107 (PMC6583976; doi:10.1371/journal.pone.0217107)
Supplement: S1 Table — (DOCX) [file pone.0217107.s004.docx]

**S1 Table.** S**ample codes of contemporary samples, provenance and collection coordinates.**

| **N°** | **Sample code** | **Provenance** | | **Coordinates** | |
| --- | --- | --- | --- | --- | --- |
|  |  | **Geographic region** | **Locality** | **Latitude** | **Longitude** |
| 1 | BQUCH0001 | Rapa Nui | Roiho | -27,112 | -109,404 |
| 2 | BQUCH0002 | Rapa Nui | Roiho | -27,112 | -109,404 |
| 3 | BQUCH0003 | Rapa Nui | Roiho | -27,112 | -109,404 |
| 4 | BQUCH0004 | Rapa Nui | Roiho | -27,112 | -109,404 |
| 5 | BQUCH0005 | Rapa Nui | Roiho | -27,112 | -109,404 |
| 6 | BQUCH0006 | Rapa Nui | Te Karava | -27,106 | -109,255 |
| 7 | BQUCH0007 | Rapa Nui | Te Karava | -27,106 | -109,255 |
| 8 | BQUCH0009 | Rapa Nui | Te Karava | -27,106 | -109,255 |
| 9 | BQUCH0010 | Rapa Nui | Te Karava | -27,106 | -109,255 |
| 10 | BQUCH0011 | Rapa Nui | Te Karava | -27,106 | -109,255 |
| 11 | BQUCH0012 | Rapa Nui | Maunga Toatoa | -27,133 | -109,300 |
| 12 | BQUCH0013 | Rapa Nui | Maunga Toatoa | -27,133 | -109,300 |
| 13 | BQUCH0014 | Rapa Nui | Maunga Toatoa | -27,133 | -109,300 |
| 14 | BQUCH0015 | Rapa Nui | Maunga Toatoa | -27,133 | -109,300 |
| 15 | BQUCH0023 | Rapa Nui | Ranu Raraku | -27,122 | -109,289 |
| 16 | BQUCH0024 | Rapa Nui | Ranu Raraku | -27,122 | -109,289 |
| 17 | BQUCH0027 | Rapa Nui | Rano Kau | -27,185 | -109,435 |
| 18 | BQUCH0028 | Rapa Nui | Rano Kau | -27,185 | -109,435 |
| 19 | BQUCH0029 | Rapa Nui | Rano Kau | -27,185 | -109,435 |
| 20 | BQUCH0030 | Rapa Nui | Rano Kau | -27,185 | -109,435 |
| 21 | BQUCH0031 | Rapa Nui | Rano Kau | -27,185 | -109,435 |
| 22 | BQUCH0032 | Rapa Nui | Rano Raraku | -27,122 | -109,289 |
| 23 | BQUCH0033 | Rapa Nui | Rano Raraku | -27,122 | -109,289 |
| 24 | BQUCH0035 | Rapa Nui | Mataveri | -27,165 | -109,435 |
| 25 | BQUCH0036 | Rapa Nui | Ara tataki Rereo | -27,165 | -109,435 |
| 26 | BQUCH0037 | Rapa Nui | Hanga Oteo | -27,060 | -109,364 |
| 27 | BQUCH0038 | Rapa Nui | Hanga Oteo | -27,060 | -109,364 |
| 28 | BQUCH0040 | Marquesas | Ua Pou | -9,365 | -140,051 |
| 29 | BQUCH0041 | Marquesas | Ua Pou | -9,365 | -140,051 |
| 30 | BQUCH0042 | Marquesas | Nuku Hiva | -9,365 | -140,051 |
| 31 | BQUCH0043 | Marquesas | Hiva Oa | -10,491 | -138,648 |
| 32 | BQUCH0045 | Marquesas | Futu Hiva | -10,513 | -138,685 |
| 33 | BQUCH0046 | Marquesas | Futu Hiva | -10,513 | -138,685 |
| 34 | BQUCH0047 | Marquesas | Futu Hiva | -10,469 | -138,665 |
| 35 | BQUCH0048 | Marquesas | Futu Hiva | -10,469 | -138,665 |
| 36 | BQUCH0049 | Marquesas | Tahuata | -9,938 | -139,108 |
| 37 | BQUCH0050 | Marquesas | Tahuata | -9,938 | -139,108 |
| 38 | BQUCH0051 | Marquesas | Tahuata | -9,952 | -139,092 |
| 39 | BQUCH0056 | Tahiti | Pirae | -17,671 | -149,427 |
| 40 | BQUCH0058 | Tahiti | Punaauia | -17,671 | -149,427 |
| 41 | BQUCH0059 | Hawaii | Hawai’i | 19,541 | -155,728 |
| 42 | BQUCH0060 | Pitcairn | Adamstown | -25,067 | -130,099 |
| 43 | BQUCH0063 | Raiatea | Raiatea* | -9,365 | -140,051 |
| 44 | BQUCH0064 | Hawaii | Hawai’i | 20,043 | -155,648 |
| 45 | BQUCH0065 | Hawaii | Hawai’i | 20,043 | -155,648 |
| 46 | BQUCH0066 | Hawaii | Hawai’i | 20,043 | -155,648 |
| 47 | BQUCH0067 | Samoa | Upolu | -14,011 | -171,779 |
| 48 | BQUCH0068 | Samoa | Upolu | -14,011 | -171,779 |
| 49 | BQUCH0074 | Samoa | Savai’i | -13,755 | -172,304 |
| 50 | BQUCH0075 | Samoa | Savai’i | -13,663 | -172,416 |

*Plant sampled in Raiatea but of reported Marquesan stock (see Seelenfreund et al. 2010)

**Continuation II. S1 Table**

| **N°** | **Sample code** | **Provenance** | | **Coordinates** | |
| --- | --- | --- | --- | --- | --- |
|  |  | **Geographic region** | **Locality** | **Latitude** | **Longitude** |
| 51 | BQUCH0077 | Samoa | Savai’i | -13,643 | -172,196 |
| 52 | BQUCH0082 | Samoa | Savai’i | -13,661 | -172,431 |
| 53 | BQUCH0083 | Samoa | Savai’i | -13,661 | -172,431 |
| 54 | BQUCH0084 | Samoa | Savai’i | -13,717 | -172,593 |
| 55 | BQUCH0085 | Samoa | Savai’i | -13,717 | -172,593 |
| 56 | BQUCH0087 | Samoa | Savai’i | -13,754 | -172,287 |
| 57 | BQUCH0088 | Samoa | Savai’i | -13,663 | -172,416 |
| 58 | BQUCH0089 | Samoa | Savai’i | -13,663 | -172,416 |
| 59 | BQUCH0090 | Samoa | Savai’i | -13,643 | -172,196 |
| 60 | BQUCH0091 | Samoa | Savai’i | -13,643 | -172,196 |
| 61 | BQUCH0092 | Samoa | Upolu | -13,906 | -171,677 |
| 62 | BQUCH0093 | Samoa | Upolu | -13,906 | -171,677 |
| 63 | BQUCH0094 | Samoa | Upolu | -13,856 | -171,750 |
| 64 | BQUCH0095 | Tonga | Tongatapu | -21,132 | -175,272 |
| 65 | BQUCH0096 | Tonga | Tongatapu | -21,132 | -175,272 |
| 66 | BQUCH0097 | Tonga | Tongatapu | -21,147 | -175,252 |
| 67 | BQUCH0098 | Tonga | Tongatapu | -21,147 | -175,252 |
| 68 | BQUCH0099 | Tonga | Tongatapu | -21,111 | -175,338 |
| 69 | BQUCH0101 | Tonga | Tongatapu | -21,077 | -175,334 |
| 70 | BQUCH0102 | Tonga | Tongatapu | -21,160 | -175,272 |
| 71 | BQUCH0103 | Tonga | Tongatapu | -21,160 | -175,272 |
| 72 | BQUCH0104 | Tonga | Tongatapu | -21,191 | -175,172 |
| 73 | BQUCH0105 | Tonga | Tongatapu | -21,139 | -175,044 |
| 74 | BQUCH0106 | Tonga | Tongatapu | -21,139 | -175,044 |
| 75 | BQUCH0107 | Tonga | Tongatapu | -21,127 | -175,108 |
| 76 | BQUCH0108 | Tonga | Tongatapu | -21,199 | -75,154 |
| 77 | BQUCH0109 | Tonga | Tongatapu | -21,199 | -75,154 |
| 78 | BQUCH0110 | Tonga | Tongatapu | -21,216 | -175,139 |
| 79 | BQUCH0111 | Tonga | Tongatapu | -21,216 | -175,139 |
| 80 | BQUCH0112 | Tonga | Tongatapu | -21,248 | -175,110 |
| 81 | BQUCH0113 | Tonga | Tongatapu | -21,214 | -175,109 |
| 82 | BQUCH0115 | Fiji | Viti Levu | -17,849 | 178,012 |
| 83 | BQUCH0116 | Fiji | Viti Levu | -17,849 | 178,012 |
| 84 | BQUCH0117 | Fiji | Viti Levu | -17,849 | 178,012 |
| 85 | BQUCH0118 | Taiwan | Da Han River | 24,247 | 120,888 |
| 86 | BQUCH0119 | Taiwan | Da Han River | 23,955 | 120,970 |
| 87 | BQUCH0120 | Taiwan | Da Du River | 24,158 | 120,494 |
| 88 | BQUCH0121 | Taiwan | Da Du River | 23,783 | 120,069 |
| 89 | BQUCH0122 | Taiwan | Jhuo Shei | 23,208 | 120,402 |
| 90 | BQUCH0123 | Taiwan | Jhuo Shei | 23,041 | 120,049 |
| 91 | BQUCH0124 | Taiwan | Zeng Wun | 22,488 | 120,232 |
| 92 | BQUCH0125 | Taiwan | Zeng Wun | 24,603 | 119,928 |
| 93 | BQUCH0127 | Taiwan | Lao Nong | 24,497 | 121,062 |
| 94 | BQUCH0128 | Taiwan | Lao Nong | 23,721 | 120,972 |
| 95 | BQUCH0129 | Taiwan | Lang Yan | 23,914 | 121,033 |
| 96 | BQUCH0130 | Taiwan | Lang Yan | 23,054 | 121,144 |
| 97 | BQUCH0131 | Taiwan | Hua Lian | 22,784 | 120,724 |
| 98 | BQUCH0132 | Taiwan | Hua Lian | 23,687 | 121,197 |
| 99 | BQUCH0133 | Taiwan | Bei Nan | 23,687 | 121,197 |
| 100 | BQUCH0134 | Chile | Santiago | -33,431 | -70,626 |

**Continuation III. S1 Table**

| **N°** | **Sample code** | **Provenance** | | **Coordinates** | |
| --- | --- | --- | --- | --- | --- |
|  |  | **Geographic region** | **Locality** | **Latitude** | **Longitude** |
| 101 | BQUCH0135 | Rapa Nui | Pu Toki Toki | -27,097 | -109,318 |
| 102 | BQUCH0136 | Pitcairn | Adamstown | -24,372 | -128,302 |
| 103 | BQUCH0137 | Taiwan | Taichung | 24,077 | 120,665 |
| 104 | BQUCH0138 | Taiwan | Taichung | 24,077 | 120,665 |
| 105 | BQUCH0139 | Taiwan | Wulai District | 24,851 | 121,549 |
| 106 | BQUCH0140 | Taiwan | Wulai District | 24,851 | 121,549 |
| 107 | BQUCH0141 | Japan | Kyoto | 35,026 | 135,781 |
| 108 | BQUCH0142 | Japan | Kyoto | 35,037 | 135,792 |
| 109 | BQUCH0143 | Japan | Kyoto | 35,037 | 135,792 |
| 110 | BQUCH0144 | Japan | Kyoto | 35,049 | 135,762 |
| 111 | BQUCH0145 | Japan | Yamashima | 35,049 | 135,762 |
| 112 | BQUCH0146 | Rapa Nui | Conaf garden | -27,165 | -109,440 |
| 113 | BQUCH0147 | Rapa Nui | Te Karava | -27,106 | -109,255 |
| 114 | BQUCH0149 | Rapa Nui | Roiho | -27,112 | -109,404 |
| 115 | BQUCH0150 | Rapa Nui | Roiho | -27,112 | -109,404 |
| 116 | BQUCH0151 | Rapa Nui | Roiho | -27,112 | -109,404 |
| 117 | BQUCH0152 | Rapa Nui | Ana te Pahu | -27,176 | -109,405 |
| 118 | BQUCH0153 | Rapa Nui | Ana te Pahu | -27,176 | -109,405 |
| 119 | BQUCH0154 | Rapa Nui | Ana te Pahu | -27,176 | -109,405 |
| 120 | BQUCH0155 | Rapa Nui | Oroi | -27,144 | -109,327 |
| 121 | BQUCH0156 | Rapa Nui | Oroi | -27,144 | -109,327 |
| 122 | BQUCH0158 | Rapa Nui | Oroi | -27,144 | -109,327 |
| 123 | BQUCH0159 | Rapa Nui | Oroi | -27,144 | -109,327 |
| 124 | BQUCH0160 | Rapa Nui | Oroi | -27,144 | -109,327 |
| 125 | BQUCH0161 | Hawaii | Oahu | 21,333 | -157,871 |
| 126 | BQUCH0162 | Hawaii | Oahu | 21,333 | -157,871 |
| 127 | BQUCH0163 | Hawaii | Oahu | 21,333 | -157,871 |
| 128 | BQUCH0164 | Hawaii | Oahu | 21,333 | -157,871 |
| 129 | BQUCH0165 | Hawaii | Oahu | 21,333 | -157,802 |
| 130 | BQUCH0166 | Hawaii | Oahu | 21,333 | -157,802 |
| 131 | BQUCH0167 | Hawaii | Oahu | 21,640 | -158,063 |
| 132 | BQUCH0168 | Hawaii | Oahu | 21,640 | -158,063 |
| 133 | BQUCH0169 | Hawaii | Oahu | 21,640 | -158,063 |
| 134 | BQUCH0170 | Hawaii | Oahu | 21,640 | -158,063 |
| 135 | BQUCH0171 | Hawaii | Oahu | 21,640 | -158,063 |
| 136 | BQUCH0172 | Hawaii | Oahu | 21,640 | -158,063 |
| 137 | BQUCH0173 | Hawaii | Oahu | 21,640 | -158,063 |
| 138 | BQUCH0174 | Hawaii | Oahu | 21,333 | -157,802 |
| 139 | BQUCH0176 | Hawaii | Hawai’i | 20,042 | -155,655 |
| 140 | BQUCH0177 | Hawaii | Hawai’i | 20,042 | -155,655 |
| 141 | BQUCH0179 | Hawaii | Hawai’i | 20,042 | -155,655 |
| 142 | BQUCH0180 | Hawaii | Hawai’i | 20,042 | -155,655 |
| 143 | BQUCH0181 | Hawaii | Hawai’i | 20,042 | -155,655 |
| 144 | BQUCH0182 | Hawaii | Hawai’i | 20,042 | -155,655 |
| 145 | BQUCH0183 | Hawaii | Hawai’i | 20,042 | -155,655 |
| 146 | BQUCH0184 | Hawaii | Hawai’i | 19,637 | -155,990 |
| 147 | BQUCH0185 | Hawaii | Hawai’i | 19,637 | -155,990 |
| 148 | BQUCH0186 | Hawaii | Hawai’i | 19,637 | -155,990 |
| 149 | BQUCH0187 | Hawaii | Hawai’i | 19,637 | -155,990 |
| 150 | BQUCH0188 | Hawaii | Hawai’i | 19,637 | -155,990 |

**Continuation IV. S1 Table**

| **N°** | **Sample code** | **Provenance** | | **Coordinates** | |
| --- | --- | --- | --- | --- | --- |
|  |  | **Geographic region** | **Locality** | **Latitude** | **Longitude** |
| 151 | BQUCH0189 | Hawaii | Hawai’i | 19,637 | -155,990 |
| 152 | BQUCH0190 | Hawaii | Oahu | 21,640 | -158,063 |
| 153 | BQUCH0191 | Hawaii | Hawai’i | 19,637 | -155,990 |
| 154 | BQUCH0192 | Hawaii | Hawai’i | 19,637 | -155,990 |
| 155 | BQUCH0193 | Hawaii | Hawai’i | 19,637 | -155,990 |
| 156 | BQUCH0194 | Hawaii | Oahu | 21,640 | -158,063 |
| 157 | BQUCH0195 | Hawaii | Oahu | 21,640 | -158,063 |
| 158 | BQUCH0201 | Vietnam | Sapa Town | 22,356 | 103,832 |
| 159 | BQUCH0202 | Vietnam | Hanoi | 22,257 | 103,887 |
| 160 | BQUCH0203 | Vietnam | Ba Vi Mountain | 21,063 | 105,366 |
| 161 | BQUCH0204 | Vietnam | Ba Vi Mountain | 21,063 | 105,366 |
| 162 | BQUCH0205 | Vietnam | Ba Vi Mountain | 21,063 | 105,366 |
| 163 | BQUCH0208 | Rapa Nui | Pu Toki Toki | -27,094 | -109,310 |
| 164 | BQUCH0209 | Rapa Nui | Pu Toki Toki | -27,094 | -109,310 |
| 165 | BQUCH0210 | Rapa Nui | Ahu Kihikihi Rau Mea | -27,123 | -109,421 |
| 166 | BQUCH0211 | Rapa Nui | Ahu Kihikihi Rau Mea | -27,123 | -109,421 |
| 167 | BQUCH0212 | Rapa Nui | Oroi | -27,134 | -109,298 |
| 168 | BQUCH0213 | Rapa Nui | Oroi | -27,134 | -109,298 |
| 169 | BQUCH0214 | Rapa Nui | Te Karava | -27,120 | -109,274 |
| 170 | BQUCH0215 | Rapa Nui | Te Karava | -27,120 | -109,274 |
| 171 | BQUCH0216 | Rapa Nui | Vai Tara Kai Ua | -27,063 | -109,350 |
| 172 | BQUCH0217 | Rapa Nui | Vai Tara Kai Ua | -27,063 | -109,350 |
| 173 | BQUCH0218 | Rapa Nui | Vai Tara Kai Ua | -27,063 | -109,350 |
| 174 | BQUCH0219 | Rapa Nui | Conaf garden | -27,164 | -109,439 |
| 175 | BQUCH0220 | Rapa Nui | Rano Kau | -27,164 | -109,439 |
| 176 | BQUCH0221 | Rapa Nui | Rano Kau | -27,181 | -109,436 |
| 177 | BQUCH0222 | Rapa Nui | Rano Kau | -27,181 | -109,436 |
| 178 | BQUCH0223 | Rapa Nui | Rano Kau | -27,181 | -109,436 |
| 179 | BQUCH0224 | Rapa Nui | Rano Kau | -27,181 | -109,436 |
| 180 | BQUCH0225 | Rapa Nui | Hanga Oteo | -27,058 | -109,376 |
| 181 | BQUCH0226 | Rapa Nui | Hanga Oteo | -27,061 | -109,361 |
| 182 | BQUCH0233 | Marquesas | Nuku Hiva | -8,828 | -140,082 |
| 183 | BQUCH0234 | Marquesas | Futu Hiva | -10,491 | -138,646 |
| 184 | BQUCH0236 | Austral Islands | Rapa | -27,618 | -144,335 |
| 185 | BQUCH0246 | Fiji | Taveuni | -16,821 | -179,871 |
| 186 | BQUCH0247 | Fiji | Taveuni | -16,821 | -179,871 |
| 187 | BQUCH0248 | Fiji | Taveuni | -16,821 | -179,871 |
| 188 | BQUCH0249 | Fiji | Taveuni | -16,821 | -179,871 |
| 189 | BQUCH0250 | Fiji | Taveuni | -16,821 | -179,871 |
| 190 | BQUCH0251 | Fiji | Taveuni | -16,821 | -179,871 |
| 191 | BQUCH0252 | Fiji | Taveuni | -16,821 | -179,871 |
| 192 | BQUCH0253 | Fiji | Taveuni | -16,821 | -179,871 |
| 193 | BQUCH0258 | Fiji | Taveuni | -16,591 | 179,735 |
| 194 | BQUCH0259 | Fiji | Taveuni | -16,591 | 179,735 |
| 195 | BQUCH0260 | Fiji | Taveuni | -16,591 | 179,735 |
| 196 | BQUCH0261 | Fiji | Taveuni | -16,591 | 179,735 |
| 197 | BQUCH0262 | Fiji | Taveuni | -16,591 | 179,735 |
| 198 | BQUCH0263 | Fiji | Taveuni | -16,591 | 179,735 |
| 199 | BQUCH0264 | Fiji | Taveuni | -16,591 | 179,735 |
| 200 | BQUCH0265 | Fiji | Taveuni | -16,591 | 179,735 |

**Continuation V. S1 Table**

| **N°** | **Sample code** | **Provenance** | | **Coordinates** | |
| --- | --- | --- | --- | --- | --- |
|  |  | **Geographic region** | **Locality** | **Latitude** | **Longitude** |
| 201 | BQUCH0266 | Fiji | Taveuni | -16,591 | 179,735 |
| 202 | BQUCH0267 | Fiji | Taveuni | -16,591 | 179,735 |
| 203 | BQUCH0268 | Wallis | Utufua | -13,342 | -176,194 |
| 204 | BQUCH0269 | Wallis | Utufua | -13,326 | -176,192 |
| 205 | BQUCH0270 | Wallis | Mata-utu | -13,301 | -176,184 |
| 206 | BQUCH0271 | Wallis | Ha’atofo | -13,344 | -176,215 |
| 207 | BQUCH0272 | Wallis | Utufua | -13,343 | -176,195 |
| 208 | BQUCH0273 | Wallis | Utufua | -13,343 | -176,195 |
| 209 | BQUCH0274 | Wallis | Utufua | -13,343 | -176,195 |
| 210 | BQUCH0275 | Fiji | Vanua Levu | -16,651 | 179,842 |
| 211 | BQUCH0276 | Fiji | Vanua Levu | -16,651 | 179,842 |
| 212 | BQUCH0277 | Fiji | Vanua Levu | -16,651 | 179,842 |
| 213 | BQUCH0278 | Fiji | Vanua Levu | -16,681 | 179,811 |
| 214 | BQUCH0279 | Fiji | Vanua Levu | -16,681 | 179,811 |
| 215 | BQUCH0280 | Fiji | Vanua Levu | -16,681 | 179,811 |
| 216 | BQUCH0281 | Fiji | Vanua Levu | -16,675 | 179,822 |
| 217 | BQUCH0282 | Fiji | Vanua Levu | -16,675 | 179,822 |
| 218 | BQUCH0283 | Fiji | Vanua Levu | -16,651 | 179,842 |
| 219 | BQUCH0284 | Fiji | Vanua Levu | -16,651 | 179,842 |
| 220 | BQUCH0285 | Fiji | Vanua Levu | -16,796 | 179,366 |
| 221 | BQUCH0286 | Fiji | Vanua Levu | -16,796 | 179,366 |
| 222 | BQUCH0287 | Fiji | Vanua Levu | -16,797 | 179,366 |
| 223 | BQUCH0288 | Fiji | Vanua Levu | -16,797 | 179,366 |
| 224 | BQUCH0289 | Fiji | Vanua Levu | -16,803 | 179,333 |
| 225 | BQUCH0290 | Fiji | Vanua Levu | -16,669 | 178,719 |
| 226 | BQUCH0291 | Fiji | Taveuni | -16,971 | 179,895 |
| 227 | BQUCH0292 | Fiji | Taveuni | -16,971 | 179,895 |
| 228 | BQUCH0293 | Fiji | Taveuni | -16,807 | 179,988 |
| 229 | BQUCH0294 | Fiji | Taveuni | -16,781 | 174,016 |
| 230 | BQUCH0295 | Fiji | Taveuni | -16,782 | 174,018 |
| 231 | BQUCH0296 | Fiji | Taveuni | -16,782 | 174,018 |
| 232 | BQUCH0297 | Fiji | Taveuni | -16,773 | 174,029 |
| 233 | BQUCH0298 | Fiji | Taveuni | 85,523 | 266,767 |
| 234 | BQUCH0299 | Fiji | Taveuni | -16,708 | 174,088 |
| 235 | BQUCH0300 | Fiji | Taveuni | -16,773 | 174,152 |
| 236 | BQUCH0301 | Fiji | Taveuni | -16,773 | 174,152 |
| 237 | BQUCH0302 | Fiji | Taveuni | -16,819 | 174,132 |
| 238 | BQUCH0303 | Fiji | Taveuni | -16,825 | 174,127 |
| 239 | BQUCH0304 | Fiji | Vatulele | -16,825 | 174,127 |
| 240 | BQUCH0305 | Fiji | Vatulele | -18,520 | 177,644 |
| 241 | BQUCH0306 | Fiji | Vatulele | -18,520 | 177,644 |
| 242 | BQUCH0307 | Fiji | Vatulele | -18,520 | 177,644 |
| 243 | BQUCH0308 | Fiji | Vatulele | -18,523 | 177,643 |
| 244 | BQUCH0309 | Fiji | Vatulele | -18,525 | 177,642 |
| 245 | BQUCH0310 | Fiji | Vatulele | -18,525 | 177,642 |
| 246 | BQUCH0311 | Fiji | Vatulele | -18,525 | 177,642 |
| 247 | BQUCH0312 | Fiji | Vatulele | -18,525 | 177,642 |
| 248 | BQUCH0313 | Fiji | Vatulele | -18,525 | 177,642 |
| 249 | BQUCH0314 | Fiji | Vatulele | -18,525 | 177,642 |
| 250 | BQUCH0315 | Fiji | Vatulele | -18,525 | 177,642 |

**Continuation VI. S1 Table**

| **N°** | **Sample code** | **Provenance** | | **Coordinates** | |
| --- | --- | --- | --- | --- | --- |
|  |  | **Geographic region** | **Locality** | **Latitude** | **Longitude** |
| 251 | BQUCH0316 | Fiji | Vatulele | -18,525 | 177,642 |
| 252 | BQUCH0317 | Fiji | Vatulele | -18,525 | 177,642 |
| 253 | BQUCH0318 | Fiji | Vatulele | -18,527 | 177,637 |
| 254 | BQUCH0319 | Fiji | Vatulele | -18,527 | 177,637 |
| 255 | BQUCH0320 | Fiji | Vatulele | -18,525 | 177,642 |
| 256 | BQUCH0321 | Fiji | Vatulele | -18,527 | 177,637 |
| 257 | BQUCH0322 | Fiji | Vatulele | -18,526 | 177,637 |
| 258 | BQUCH0323 | Fiji | Viti Levu | -18,090 | 178,451 |
| 259 | BQUCH0324 | Fiji | Viti Levu | -18,090 | 178,451 |
| 260 | BQUCH0325 | Fiji | Viti Levu | -18,145 | 178,433 |
| 261 | BQUCH0326 | Fiji | Viti Levu | -18,128 | 178,449 |
| 262 | BQUCH0327 | Fiji | Viti Levu | -18,128 | 178,449 |
| 263 | BQUCH0328 | Wallis | Utufua | -13,329 | -176,192 |
| 264 | BQUCH0329 | Wallis | Utufua | -13,309 | -176,189 |
| 265 | BQUCH0330 | Wallis | Mata-Utu | -13,276 | -176,172 |
| 266 | BQUCH0331 | Wallis | Mata-Utu | -13,301 | -176,184 |
| 267 | BQUCH0332 | Wallis | Ha’atofo | -13,314 | -176,233 |
| 268 | BQUCH0333 | Wallis | Ha’atofo | -13,344 | -176,215 |
| 269 | BQUCH0338 | Tonga | Tongatapu | -21,134 | -175,200 |
| 270 | BQUCH0339 | Tonga | Tongatapu | -21,122 | -175,221 |
| 271 | BQUCH0340 | Tonga | Tongatapu | -21,150 | -175,193 |
| 272 | BQUCH0341 | Tonga | Tongatapu | -21,163 | -175,232 |
| 273 | BQUCH0342 | Tonga | Tongatapu | -21,193 | -175,132 |
| 274 | BQUCH0343 | Tonga | Tongatapu | -20,885 | -175,235 |
| 275 | BQUCH0344 | Tonga | Tongatapu | -21,156 | -175,232 |
| 276 | BQUCH0345 | Tonga | Tongatapu | -21,149 | -175,220 |
| 277 | BQUCH0346 | Tonga | Tongatapu | -21,138 | -175,227 |
| 278 | BQUCH0347 | Tonga | Tongatapu | -21,135 | -175,267 |
| 279 | BQUCH0348 | Tonga | Tongatapu | -21,134 | -175,280 |
| 280 | BQUCH0349 | Tonga | Tongatapu | -21,125 | -175,323 |
| 281 | BQUCH0350 | Tonga | Tongatapu | -21,068 | -175,326 |
| 282 | BQUCH0351 | Tonga | Tongatapu | -21,087 | -175,339 |
| 283 | BQUCH0352 | Tonga | Tongatapu | -21,148 | -175,328 |
| 284 | BQUCH0353 | Tonga | Tongatapu | -21,156 | -175,284 |
| 285 | BQUCH0354 | Tonga | Tongatapu | -21,174 | -175,249 |
| 286 | BQUCH0355 | Tonga | Vava'u | -18,652 | -173,984 |
| 287 | BQUCH0356 | Tonga | Vava'u | -18,652 | -173,983 |
| 288 | BQUCH0357 | Tonga | Vava'u | -18,578 | -173,948 |
| 289 | BQUCH0358 | Tonga | Vava'u | -18,644 | -173,920 |
| 290 | BQUCH0359 | Tonga | Vava'u | -18,632 | -168,016 |
| 291 | BQUCH0360 | Tonga | Vava'u | -18,630 | -168,012 |
| 292 | BQUCH0361 | Tonga | Eua | -21,393 | -174,940 |
| 293 | BQUCH0362 | Tonga | Eua | -21,440 | -174,926 |
| 294 | BQUCH0363 | Tonga | Eua | -21,440 | -174,926 |
| 295 | BQUCH0364 | Tonga | Eua | -21,448 | -174,915 |
| 296 | BQUCH0365 | Tonga | Eua | -21,451 | -174,912 |
| 297 | BQUCH0366 | Tonga | Eua | -21,424 | -174,944 |
| 298 | BQUCH0367 | Tonga | Eua | -21,364 | -174,955 |
| 299 | BQUCH0368 | Tonga | Eua | -21,308 | -174,927 |
| 300 | BQUCH0369 | Tonga | Eua | -21,337 | -174,952 |

**Continuation VII. S1 Table**

| **N°** | **Sample code** | **Provenance** | | **Coordinates** | |
| --- | --- | --- | --- | --- | --- |
|  |  | **Geographic region** | **Locality** | **Latitude** | **Longitude** |
| 301 | BQUCH0370 | Tonga | Eua | -21,337 | -174,952 |
| 302 | BQUCH0428 | China | Ruayuan Yao | 24,948 | 113,420 |
| 303 | BQUCH0429 | China | Ruayuan Yao | 24,948 | 113,420 |
| 304 | BQUCH0430 | China | Ruayuan Yao | 24,948 | 113,420 |
| 305 | BQUCH0431 | China | Guandong | 22,456 | 113,550 |
| 306 | BQUCH0432 | Tahiti | Puna’auia | -17,632 | -149,614 |
| 307 | BQUCH0433 | Tahiti | Puna’auia | -17,632 | -149,614 |
| 308 | BQUCH0434 | Tahiti | Puna’auia | -17,632 | -149,614 |
| 309 | BQUCH0435 | Tahiti | Paea | -17,702 | -149,582 |
| 310 | BQUCH0439 | Austral Islands | Rapa | -27,619 | -144,336 |
| 311 | BQUCH0442 | New Caledonia | Nouméa | -22,165 | 166,663 |
| 312 | BQUCH0443 | New Caledonia | Nouméa | -22,165 | 166,663 |
| 313 | BQUCH0444 | New Caledonia | Nouméa | -22,165 | 166,663 |
