# Supplementary material for: Human mediated translocation of Pacific paper mulberry [Broussonetia papyrifera (L.) L’Hér. ex Vent. (Moraceae)]: Genetic evidence of dispersal routes in Remote Oceania
Source: PLoS One. 2019 Jun 19;14(6):e0217107. doi: 10.1371/journal.pone.0217107 (PMC6583976; doi:10.1371/journal.pone.0217107)
Supplement: S2 Table — (DOCX) [file pone.0217107.s005.docx]

**S2 Table. Herbarium samples included in this study with codes, field collection number, geographic origin, collectors and year of collection**

| **N°** | **Herbarium code** | **Provenance** | | **Collector** | **Collection number** | **Year** |
| --- | --- | --- | --- | --- | --- | --- |
|  |  | **Geographic region** | **Locality** |  |  |  |
| 1 | SG0005091 | Chile | Santiago | F. Phillipi | 6605 | 1882 |
| 2 | BISH58376 | Hawaii | Oahu | W.A. Bryan | - | 1903 |
| 3 | BISH451764 | Hawaii | Kauai | J.F.C. Rock | 5848 | 1909 |
| 4 | BISH451765 | Hawaii | Kauai | J.F.C. Rock | 1585 | 1909 |
| 5 | BISH451766 | Hawaii | Kauai | J.F.C. Rock | 1756 | 1909 |
| 6 | BISH58346 | Hawaii | Hawai’i | J.F.C. Rock | 3663 | 1909 |
| 7 | BISH58360 | Hawaii | Kauai | J.F.C. Rock | 2380 | 1909 |
| 8 | BISH58362 | Hawaii | Kauai | J.F.C. Rock | 2373 | 1909 |
| 9 | BISH58363 | Hawaii | Kauai | J.F.C. Rock | 2548 | 1909 |
| 10 | SG0058300 | Rapa Nui | Rapa Nui | F. Fuentes | - | 1911 |
| 11 | SGO058271 | Rapa Nui | Rapa Nui | F. Fuentes | - | 1911 |
| 12 | BISH57308 | Hawaii | Niihau | J.F.G. Stokes | - | 1912 |
| 13 | BISH58378 | Hawaii | Lanai | C.N. Forbes | 92 | 1913 |
| 14 | BISH161285 | Rapa Nui | Rano Kau | Skottsberg, C.J.F. | - | 1917 |
| 15 | BISH58368 | Hawaii | Oahu | J.F.C. Rock | 17052 | 1918 |
| 16 | BISH161281 | Marquesas | Nuku Hiva | F.B.H. Brown & E.D.W. Brown | 664 | 1921 |
| 17 | BISH161290 | Austral Islands | Rapa | A.M. Stokes | 24 | 1921 |
| 18 | BISH161292 | Austral Islands | Rurutu | J.F.G. Stokes | 136 | 1921 |
| 19 | BISH161293 | Austral Islands | Rapa | J.F.G. Stokes | 412 | 1921 |
| 20 | BISH161294 | Austral Islands | Rapa | J.F.G. Stokes | 412 | 1921 |
| 21 | BISH161296 | Austral Islands | Rapa | J.F.G. Stokes | 216 | 1921 |
| 22 | BISH751633 | Austral Islands | Rapa | A.M. & J.F.G. Stokes | 140 | 1921 |
| 23 | BISH751636 | Austral Islands | Rapa | A.M .& J.F.G. Stokes | 216 | 1921 |
| 24 | BISH161324 | Fiji | Viti Levu Group Ovalau | E.H. Bryan Jr. | 474 | 1924 |
| 25 | BISH58357 a | Hawaii | Hawai’i South Kona | K.P. Emory | 1272 | 1924 |
| 26 | BISH58357 b | Hawaii | Hawai’i South Kona | K. P. Emory | 1272 | 1924 |
| 27 | BISH58358 a | Hawaii | Hawai’i | K.P. Emory | 1 | 1924 |
| 28 | BISH58358 b | Hawaii | Hawai’i | K.P. Emory | 1 | 1924 |
| 29 | BISH668629 | Hawaii | Hawaii’ | M.C. Neal | 1274 | 1924 |
| 30 | BISH763194 | Hawaii | Hawai’i | M.C. Neal | 1273 | 1924 |
| 31 | BISH750662 | American Samoa | Olosega | D. W. Garber | 1071 | 1925 |
| 32 | BISH58370 | Hawaii | Lanai | G.C. Munro | 136 | 1927 |
| 33 | BISH58352 | Hawaii | Molokai | O. Degener | 8688 | 1928 |
| 34 | BISH58385 | Hawaii | Molokai | O. Degener | 8688 | 1928 |
| 35 | BISH58386 | Hawaii | Molokai | O. Degener | 8637 | 1928 |
| 36 | BISH161280 | Cook Islands | Rarotonga | Wilder, G.P. | 899 | 1929 |
| 37 | BISH58381 | Hawaii | Oahu | O. Degener | 8686 | 1929 |
| 38 | BISH58387 | Hawaii | Oahu | O. Degener | 8682 | 1930 |
| 39 | BISH445703 | Hawaii | Oahu | E. Christophersen | 1628 | 1931 |

a, b Two samples taken from the same sheet and labeled as different specimens

**Continuation II S2 Table**

| **N°** | **Herbarium code** | **Provenance** | | **Collector** | **Collection number** | **Year** |
| --- | --- | --- | --- | --- | --- | --- |
|  |  | **Geographic region** | **Locality** |  |  |  |
| 40 | BISH58384 | Hawaii | Oahu | O. Degener | 8683 | 1931 |
| 41 | BISH58355 | Hawaii | Oahu | E. Christophersen | 3719 | 1932 |
| 42 | BISH58382 | Hawaii | Oahu | O. Degener | 8685 | 1932 |
| 43 | BISH161287 | Pitcairn | Adamstown | Chapin, J.P. | 965 | 1934 |
| 44 | BISH161288 | Pitcairn | Adamstown | H. St. John | 15032 | 1934 |
| 45 | BISH161326 | Fiji | Fiji east coast | A.C. Smith | 1097 | 1934 |
| 46 | BISH99313 | Hawaii | Oahu | M.L. Grant | 7129 | 1934 |
| 47 | BISH161284 | Rapa Nui | Rano Kau | Chapin, J.P. | 1009 | 1935 |
| 48 | BISH161323 | New Guinea | Near Ekua | B. Blackwood | 260 | 1937 |
| 49 | BISH58371 | Hawaii | Oahu | E.L. Caum | - | 1937 |
| 50 | BISH161277 | American Samoa | Ta'u Is. | T.G. Yuncker | 9204 | 1939 |
| 51 | BISH161275 | Niue | Niue | Not indicated | 10114 | 1940 |
| 52 | BISH161276 | Niue | Niue | Not indicated | 10114 | 1940 |
| 53 | BISH415077 | Hawaii | Niihau | H. St.John | 23638 | 1949 |
| 54 | BISH161279 | Tonga | Eua | T.G. Yuncker | 15471 | 1953 |
| 55 | BISH664608 | Pitcairn | Pitcairn | Lintott, W.H | 81 | 1957 |
| 56 | AK116673 | N. Guinea | Kaironk Valley | R.N.H. Bulmer | 219 | 1964 |
| 57 | BISH32928 | Fiji | Yacata | D. Koroiveibau | 15559 | 1968 |
| 58 | BISH757984 | Fiji | Yacata | D. Koroiveibau | 15560 | 1968 |
| 59 | SG0129525 | Rapa Nui | Rapa Nui | F. Sudzuki | - | 1971 |
| 60 | BISH418270 | Futuna | Alo District | P. Kirch | 20 | 1974 |
| 61 | BISH416666 | Solomon Is. | Guadalcanal | N.L.H. Krauss | 1445 | 1977 |
| 62 | BISH505999 | Solomon Is. | Guadalcanal | N.L.H. Krauss | 1801 | 1985 |
| 63 | AK214298 | Solomon Is. | Guadalcanal | R.O. Gardener | 7418 | 1993 |
| 64 | SGO141121 | China | Xining Co | Luo Lin Bo | 633 | 1995 |
| 65 | BISH709092 | Marquesas | Ua Huka | J.Y. Meyer | 2000-094 | 2003 |
| 66 | AK295889 | New Zealand | Auckland | D.S. McKenzie | 06.01 | 2006 |
| 67 | AK296981 | New Zealand | Auckland | P.J. de Lange | 6642 | 2006 |

a, b Two samples taken from the same sheet and labeled as different specimens

BISH: Herbarium Pacificum, B.P. Bishop Museum, Honolulu, Hawaii, USA; SGO: Herbario Nacional, National Museum of Natural History in Santiago, Chile; AK: The Auckland Museum Herbarium, Auckland War Memorial Museum, Auckland, New Zealand.
