# Supplementary material for: Human mediated translocation of Pacific paper mulberry [Broussonetia papyrifera (L.) L’Hér. ex Vent. (Moraceae)]: Genetic evidence of dispersal routes in Remote Oceania
Source: PLoS One. 2019 Jun 19;14(6):e0217107. doi: 10.1371/journal.pone.0217107 (PMC6583976; doi:10.1371/journal.pone.0217107)
Supplement: S3 Table — (DOCX) [file pone.0217107.s006.docx]

**S3 Table. Sequences and annealing temperature of primers used in this work**

| **Marker** | **Annealing temperature** | **Sequence** |
| --- | --- | --- |
| ITS-1 | 60 °C | Fw: 5’-GGA AGG AGA AGT CGT AAC AAG G-3’ |
|  |  | Rev:5’-GCA ATT CAC ACC AAG TAT CGC-3’ |
| *Male Marker* | 55 °C | Fw: 5'-AGC CCC TTT GGA TCG CGA CTT AGA A-3' |
|  |  | RS18: 5'- TGT CAA CGT CAT CAT CGT CGT CGT-3' |
|  |  | RL 5’- CTG GAC AAG ACC AAC TTT GAA TCC G-3’ |
| *nhdF-rpl32* | 55- 65 °C for one min | Fw: 5'- GAA AGG TAT KAT CCA YGM ATA TT-3' |
|  |  | Rev: 5'- CCA ATA TCC CTT YYT TTT CCA A-3' |
| Bro 07 | 55 °C | Fw: 5' - GCT TTC TGT ACC CCC AA ATG - 3' |
|  |  | Rev: 5' - TTC TTT AGC TCG AGG GCG TA - 3' |
| Bro 08 | 55 °C | Fw: 5’- TAG ACC CAC CTC CCA AAC AG-3’ |
|  |  | Rev:5’- ATT CGG AGG CAT CTG AGA GA-3’ |
| Bro 13 | 55°C | Fw: 5' - AGA GAG GGA CAA CAT CAA CGA - 3' |
|  |  | Rev: 5' - TGG TCT GTG ACC CAT TTC TTC - 3' |
| Bro 15 | 56°C | Fw: 5’- CCG TAG CGA TCT TCC AGA AA-3’ |
|  |  | Rev:5’- AAA GAA ATG GCG GAA ATG TG-3’ |
| Bropap 02214 | 55°C | Fw: 5’- CGG AAG AGA GAG ATT ACA AAC TAG C-3’ |
|  |  | Rev:5’- ACT TGA CCA TTC TAA GCA AGA CC-3’ |
| Bropap 02801 | 56°C | Fw: 5’- GAC ATC TCA TAA AAT GTT TAA ATC CAG-3’ |
|  |  | Rev:5’- AAT TGC CCT GTA GCA TTC CG-3’ |
| Bropap 20558 | 55°C | Fw: 5’- TCC ACC ATC CAA CGA TGA AG-3’ |
|  |  | Rev: 5’- GGG CTA AAC TAC CTT GTC CG-3’ |
| Bropap 26985 | 56°C | Fw: 5’- AGA ATC ACC ACT CTC CCT TGG-3’ |
|  |  | Rev: 5’-TGG TTT GCT TCA TTC AAA AAG TG-3’ |
| Bropap 25444 | 55°C | Fw: 5' - TCA CAC TTA CAC ACG GAG GG - 3' |
|  |  | Rev: 5' - GGT ACG TAA TTC CCA CCA CC - 3' |
| Bropap 30248 | 56°C | Fw: 5’- AGA GCA GGG CAA GCA ATA TC-3’ |
|  |  | Rev: 5’- GGT CAT CCA TTT GTC TGA ACC TC-3’ |
