# Supplementary material for: Human mediated translocation of Pacific paper mulberry [Broussonetia papyrifera (L.) L’Hér. ex Vent. (Moraceae)]: Genetic evidence of dispersal routes in Remote Oceania
Source: PLoS One. 2019 Jun 19;14(6):e0217107. doi: 10.1371/journal.pone.0217107 (PMC6583976; doi:10.1371/journal.pone.0217107)
Supplement: S4 Table — (DOCX) [file pone.0217107.s007.docx]

**S4 Table. cpDNA haplotypes found on different islands and number of samples per haplotype**

|  | **Locality** | **N of Samples** | | **Number of Samples *per* Haplotype** | | | | |
| --- | --- | --- | --- | --- | --- | --- | --- | --- |
|  |  | **Leaf** | **Herbarium** |  |  |  |  |  |
|  |  |  |  | **cp17** | **cp41** | **cp49** | **cp50** | **Asian haplotype** |
| Near Oceania | Solomon Is. | - | 2 | - | - | - | - | 2 |
|  | New Guinea | - | 2 | 2 | - | - | - | - |
| West Remote Oceania | Fiji | 6 | 4 | 10 | - | - | - | - |
|  | Tonga | 6 | 1 | 7 | - | - | - | - |
|  | Futuna | - | 1 | 1 | - | - | - | - |
|  | Samoa | 4 | 1 | 5 | - | - | - | - |
|  | Wallis | 1 | - | 1 | - | - | - | - |
|  | New Caledonia | 1 | - | 1 | - | - | - | - |
| East Remote Oceania | Marquesas | 4 | 1 | 4 | - | - | 1 | - |
|  | Cook Is. | - | 1 | 1 | - | - | - | - |
|  | Niue | - | 2 | 2 | - | - | - | - |
|  | Austral Islands | 1 | 6 | 7 | - | - | - | - |
|  | Pitcairn | - | 3 | 3 | - | - | - | - |
|  | Tahíti | 2 | - | 2 | - | - | - | - |
|  | Rapa Nui | 7 | 4 | 10 | - | 1 | - | - |
|  | Hawaii | 3 | 29 | 28 | 3 | - | - | 1 |
|  | **Total** | **35** | **57** | **84** | **3** | **1** | **1** | **3** |
