# Supplementary material for: Human mediated translocation of Pacific paper mulberry [Broussonetia papyrifera (L.) L’Hér. ex Vent. (Moraceae)]: Genetic evidence of dispersal routes in Remote Oceania
Source: PLoS One. 2019 Jun 19;14(6):e0217107. doi: 10.1371/journal.pone.0217107 (PMC6583976; doi:10.1371/journal.pone.0217107)
Supplement: S5 Table — (DOCX) [file pone.0217107.s008.docx]

**S5 Table. Private alleles found in Remote Oceania genotypes for each microsatellite marker**

| **Locality** | ***Locus*** | **Allele** |
| --- | --- | --- |
| Lanai (Hawaii) | Bro 08 | 182 |
| Cook Is. | Bro 15 | 206 |
| New Caledonia | Bropap 2801 | 180 |
|  | Bropap 25444 | 185 |
|  | Bropap 25444 | 187 |
| Nuku Hiva (Marquesas Is) | Bro 08 | 192  211* |
| Oahu_f (Hawaii) | Bro 08 | 209 |
|  | Bro 13 | 222 |
|  | Bro 15 | 228 |
|  | Bropap 2214 | 219 |
|  | Bropap 2214 | 229 |
|  | Bropap 2214 | 236 |
|  | Bropap 2214 | 244 |
|  | Bropap 2801 | 145 |
|  | Bropap 2801 | 177 |
|  | Bropap 20558 | 206 |
|  | Bropap 20558 | 219 |
|  | Bropap 20558 | 223 |
|  | Bropap 25444 | 177 |
|  | Bropap 25444 | 180 |
|  | Bropap 26985 | 173 |
|  | Bropap 30248 | 97 |
| Rapa (Austral Is.) | Bro 08 | 220  239* |
| Rapa Nui | Bro 13 | 227 |
|  | Bropap 25444 | 190 |
| Savai’i (Samoa) | Bropap 2801 | 160 |
| American Samoa | Bropap 2801 | 179* |
| Tongatapu (Tonga) | Bro 08 | 198 |
| Vanua Levu (Fiji) | Bropap 25444 | 176 |
| Viti Levu (Fiji) | Bropap 25444 | 181 |
| Yacata (Fiji) | Bropap 2214 | 235 |
|  | Bropap 20558 | 225 |

*****Alleles found only in herbarium samples
