# Supplementary material for: Human mediated translocation of Pacific paper mulberry [Broussonetia papyrifera (L.) L’Hér. ex Vent. (Moraceae)]: Genetic evidence of dispersal routes in Remote Oceania
Source: PLoS One. 2019 Jun 19;14(6):e0217107. doi: 10.1371/journal.pone.0217107 (PMC6583976; doi:10.1371/journal.pone.0217107)
Supplement: S6 Table — (DOCX) [file pone.0217107.s009.docx]

**S6 Table. Allele frequencies found on different islands in Near and Remote Oceania for each microsatellite**

| ***Locus*** | **Geographic location** | **A. Samoa** | **Austral Is.** | | **Cook Is.** | **Fiji** | | | | | **Futuna** | **Hawaii** | | | | | | **Marquesas Is.** | | | | | | **N. Caledonia** | **N. Guinea** | **Niue** | **Pitcairn** | **Rapa Nui** | **Samoa** | | **Tahiti** | **Tonga** | | | **Wallis** |
| --- | --- | --- | --- | --- | --- | --- | --- | --- | --- | --- | --- | --- | --- | --- | --- | --- | --- | --- | --- | --- | --- | --- | --- | --- | --- | --- | --- | --- | --- | --- | --- | --- | --- | --- | --- |
|  | **Locality** | A. Samoa | Rapa | Rurutu | Cook Is. | Taveuni | Vanua Levu | Vatulele | Viti Levu | Yacata | Futuna | Hawai'i f | Kauai | Lanai | Molokai | Oahu f | Ni'iahu | Fatu Hiva | Hiva Oa | Nuku Hiva | Tahuata | Ua Huka | Ua Pou | N. Caledonia | N. Guinea | Niue | Pitcairn | Rapa Nui | Savai'i | Upolu | Tahiti | Eua | Tongatapu | Vava'u | Wallis |
|  | **Allele/ N of samples** | 1 | 8 | 1 | 1 | 31 | 16 | 19 | 10 | 2 | 1 | 18 | 6 | 2 | 3 | 18 | 2 | 5 | 2 | 3 | 3 | 1 | 2 | 3 | 2 | 2 | 5 | 65 | 13 | 5 | 6 | 11 | 35 | 6 | 13 |
| **Bro 08** | 182 |  |  |  |  |  |  |  |  |  |  |  |  | **0,25** |  |  |  |  |  |  |  |  |  |  |  |  |  |  |  |  |  |  |  |  |  |
|  | 192 |  |  |  |  |  |  |  |  |  |  |  |  |  |  |  |  |  |  | **0,17** |  |  |  |  |  |  |  |  |  |  |  |  |  |  |  |
|  | 198 |  |  |  |  |  |  |  |  |  |  |  |  |  |  |  |  |  |  |  |  |  |  |  |  |  |  |  |  |  |  |  | **0,01** |  |  |
|  | 202 | 0,50 | 0,50 | 0,50 | 0,50 | 0,50 | 0,50 | 0,50 | 0,50 | 0,50 | 0,50 | 0,47 | 0,58 | 0,25 | 0,50 | 0,33 | 0,50 | 0,50 | 0,50 | 0,50 | 0,67 | 0,50 | 0,50 | 0,50 | 0,50 | 0,50 | 0,50 | 0,48 | 0,50 | 0,50 | 0,50 | 0,05 | 0,47 | 0,50 | 0,50 |
|  | 204 |  |  |  |  |  |  |  |  |  |  |  |  |  |  | 0,03 |  |  |  |  |  |  |  |  |  |  |  | 0,02 |  |  |  | 0,45 | 0,01 |  |  |
|  | 206 | 0,50 | 0,25 | 0,50 | 0,50 | 0,50 | 0,50 | 0,50 | 0,50 | 0,50 | 0,50 | 0,50 | 0,42 | 0,50 | 0,50 | 0,42 | 0,50 | 0,50 | 0,25 | 0,33 | 0,33 | 0,50 | 0,50 | 0,50 | 0,50 | 0,50 | 0,50 | 0,47 | 0,50 | 0,50 | 0,50 | 0,45 | 0,49 | 0,50 | 0,50 |
|  | 208 |  |  |  |  |  |  |  |  |  |  | 0,03 |  |  |  | 0,19 |  |  | 0,25 |  |  |  |  |  |  |  |  | 0,02 |  |  |  |  |  |  |  |
|  | 209 |  |  |  |  |  |  |  |  |  |  |  |  |  |  | **0,03** |  |  |  |  |  |  |  |  |  |  |  |  |  |  |  |  |  |  |  |
|  | 210 |  |  |  |  |  |  |  |  |  |  |  |  |  |  |  |  |  |  |  |  |  |  |  |  |  |  | 0,01 |  |  |  | 0,05 | 0,01 |  |  |
|  | 220 |  | **0,25** |  |  |  |  |  |  |  |  |  |  |  |  |  |  |  |  |  |  |  |  |  |  |  |  |  |  |  |  |  |  |  |  |
| **Bro 13** | 222 |  |  |  |  |  |  |  |  |  |  |  |  |  |  | **0,06** |  |  |  |  |  |  |  |  |  |  |  |  |  |  |  |  |  |  |  |
|  | 226 | 1,00 | 1,00 | 1,00 | 1,00 | 1,00 | 1,00 | 1,00 | 1,00 | 1,00 | 1,00 | 1,00 | 1,00 | 1,00 | 1,00 | 0,94 | 1,00 | 1,00 | 1,00 | 1,00 | 1,00 | 1,00 | 1,00 | 1,00 | 1,00 | 1,00 | 1,00 | 0,99 | 1,00 | 1,00 | 1,00 | 1,00 | 1,00 | 1,00 | 1,00 |
|  | 227 |  |  |  |  |  |  |  |  |  |  |  |  |  |  |  |  |  |  |  |  |  |  |  |  |  |  | **0,01** |  |  |  |  |  |  |  |
| **Bro 15** | 206 |  |  |  | **0,50** |  |  |  |  |  |  |  |  |  |  |  |  |  |  |  |  |  |  |  |  |  |  |  |  |  |  |  |  |  |  |
|  | 210 | 0,50 | 0,50 | 0,50 | 0,50 | 0,50 | 0,50 | 0,50 | 0,50 | 0,50 | 0,50 | 0,50 | 0,58 | 0,50 | 0,50 | 0,53 | 0,50 | 0,50 | 0,50 | 0,50 | 0,50 | 0,50 | 0,50 | 0,50 | 0,50 | 0,50 | 0,50 | 0,50 | 0,50 | 0,50 | 0,50 | 0,50 | 0,50 | 0,50 | 0,50 |
|  | 214 | 0,50 |  |  |  | 0,50 | 0,50 | 0,50 | 0,50 | 0,50 | 0,50 | 0,08 |  |  |  | 0,19 |  |  | 0,50 | 0,17 | 0,17 |  |  | 0,50 | 0,50 |  | 0,50 | 0,50 | 0,50 | 0,50 | 0,17 | 0,45 | 0,50 | 0,50 | 0,50 |
|  | 221 |  | 0,50 | 0,50 |  |  |  |  |  |  |  | 0,42 | 0,42 | 0,50 | 0,50 | 0,25 | 0,50 | 0,50 |  | 0,33 | 0,33 | 0,50 | 0,50 |  |  | 0,50 |  |  |  |  | 0,33 | 0,05 |  |  |  |
|  | 228 |  |  |  |  |  |  |  |  |  |  |  |  |  |  | **0,03** |  |  |  |  |  |  |  |  |  |  |  |  |  |  |  |  |  |  |  |
| **Bropap 02214** | 219 |  |  |  |  |  |  |  |  |  |  |  |  |  |  | **0,03** |  |  |  |  |  |  |  |  |  |  |  |  |  |  |  |  |  |  |  |
|  | 229 |  |  |  |  |  |  |  |  |  |  |  |  |  |  | **0,03** |  |  |  |  |  |  |  |  |  |  |  |  |  |  |  |  |  |  |  |
|  | 235 |  |  |  |  |  |  |  |  | **0,25** |  |  |  |  |  |  |  |  |  |  |  |  |  |  |  |  |  |  |  |  |  |  |  |  |  |
|  | 236 |  |  |  |  |  |  |  |  |  |  |  |  |  |  | **0,03** |  |  |  |  |  |  |  |  |  |  |  |  |  |  |  |  |  |  |  |
|  | 239 |  |  |  |  |  |  |  | 0,05 |  |  |  |  |  |  | 0,03 |  |  |  |  |  |  |  |  |  |  |  |  |  |  |  |  |  |  |  |
|  | 241 |  | 0,44 | 0,50 | 0,50 | 0,48 | 0,41 | 0,50 | 0,45 | 0,25 | 0,50 | 0,47 | 0,50 | 0,50 | 0,67 | 0,44 | 0,50 | 0,50 |  | 0,33 | 0,33 | 0,50 | 0,50 |  | 0,75 | 0,50 | 0,50 |  | 0,46 | 0,50 | 0,33 | 0,50 | 0,49 | 0,50 | 0,50 |
|  | 243 | 0,50 |  |  | 0,00 | 0,02 |  |  |  | 0,25 |  | 0,03 |  |  |  |  |  |  | 0,50 | 0,17 | 0,17 |  |  | 0,50 |  |  |  | 0,48 | 0,04 |  | 0,17 |  | 0,01 |  |  |
|  | 244 |  |  |  |  |  |  |  |  |  |  |  |  |  |  | **0,03** |  |  |  |  |  |  |  |  |  |  |  |  |  |  |  | 0,00 |  |  |  |
|  | 247 | 0,00 |  |  | 0,00 |  | 0,09 |  |  |  |  |  |  |  |  |  |  |  |  |  |  |  |  |  |  |  |  | 0,02 |  |  |  |  |  |  |  |
|  | 249 |  | 0,56 | 0,50 | 0,50 | 0,47 | 0,41 | 0,50 | 0,40 |  | 0,50 | 0,36 | 0,50 | 0,25 | 0,33 | 0,42 | 0,50 | 0,50 | 0,50 | 0,17 | 0,50 | 0,50 | 0,50 | 0,50 |  | 0,50 | 0,50 | 0,29 | 0,46 | 0,30 | 0,42 | 0,50 | 0,50 | 0,50 | 0,50 |
|  | 251 | 0,50 |  |  |  | 0,02 | 0,09 |  | 0,10 | 0,25 |  | 0,11 |  | 0,25 |  |  |  |  |  | 0,17 |  |  |  |  | 0,25 |  |  | 0,21 | 0,04 | 0,20 |  |  |  |  |  |
|  | 253 |  |  |  |  | 0,02 |  |  |  |  |  | 0,03 | 0,00 |  |  |  |  |  |  | 0,17 |  |  |  |  |  |  |  |  |  |  | 0,08 |  |  |  |  |

**Continuation II S6 Table**

| ***Locus*** | **Geographic location** | **A. Samoa** | **Austral Is.** | | **Cook Is.** | **Fiji** | | | | | **Futuna** | **Hawaii** | | | | | | **Marquesas Is.** | | | | | | **N. Caledonia** | **N. Guinea** | **Niue** | **Pitcairn** | **Rapa Nui** | **Samoa** | | **Tahiti** | **Tonga** | | | **Wallis** |
| --- | --- | --- | --- | --- | --- | --- | --- | --- | --- | --- | --- | --- | --- | --- | --- | --- | --- | --- | --- | --- | --- | --- | --- | --- | --- | --- | --- | --- | --- | --- | --- | --- | --- | --- | --- |
|  | **Locality** | A. Samoa | Rapa | Rurutu | Cook Is. | Taveuni | Vanua Levu | Vatulele | Viti Levu | Yacata | Futuna | Hawai'i f | Kauai | Lanai | Molokai | Oahu f | Ni'iahu | Fatu Hiva | Hiva Oa | N. Hiva | Tahuata | Ua Huka | Ua Pou | N. Caledonia | N. Guinea | Niue | Pitcairn | Rapa Nui | Savai'i | Upolu | Tahiti | Eua | Tongatapu | Vava'u | Wallis |
| **Bropap 02801** | 145 |  |  |  |  |  |  |  |  |  |  |  |  |  |  | **0,03** |  |  |  |  |  |  |  |  |  |  |  |  |  |  |  |  |  |  |  |
|  | 149 | 0,50 | 0,50 | 0,50 | 0,50 | 0,50 | 0,50 | 0,50 | 0,50 | 0,50 | 0,50 | 0,50 | 0,58 | 0,50 | 0,50 | 0,44 | 0,50 | 0,50 | 0,50 | 0,50 | 0,50 | 0,50 | 0,50 | 0,50 | 0,50 | 0,50 | 0,50 | 0,50 | 0,50 | 0,50 | 0,50 | 0,50 | 0,50 | 0,50 | 0,50 |
|  | 159 |  |  |  |  |  |  |  |  |  |  | 0,06 |  |  |  | 0,17 |  |  |  |  |  |  |  |  |  |  |  |  |  |  |  |  |  |  |  |
|  | 160 |  |  |  |  |  |  |  |  |  |  |  |  |  |  |  |  |  |  |  |  |  |  |  |  |  |  |  | **0,04** |  |  |  |  |  |  |
|  | 170 |  | 0,50 | 0,50 | 0,50 | 0,02 | 0,09 |  | 0,05 | 0,25 |  | 0,42 | 0,42 | 0,50 | 0,50 | 0,31 | 0,50 | 0,50 | 0,50 | 0,50 | 0,50 | 0,50 | 0,50 |  | 0,50 |  | 0,50 | 0,50 |  |  | 0,17 | 0,05 | 0,01 |  |  |
|  | 172 | 0,50 |  |  |  | 0,48 | 0,41 | 0,50 | 0,45 | 0,25 | 0,50 | 0,03 |  |  |  | 0,03 |  |  |  |  |  |  |  |  |  | 0,50 |  |  | 0,46 | 0,50 | 0,33 | 0,45 | 0,49 | 0,50 | 0,50 |
|  | 177 |  |  |  |  |  |  |  |  |  |  |  |  |  |  | **0,03** |  |  |  |  |  |  |  |  |  |  |  |  |  |  |  |  |  |  |  |
|  | 180 |  |  |  |  |  |  |  |  |  |  |  |  |  |  |  |  |  |  |  |  |  |  | **0,50** |  |  |  |  |  |  |  |  |  |  |  |
| **Bropap 20558** | 206 |  |  |  |  |  |  |  |  |  |  |  |  |  |  | **0,03** |  |  |  |  |  |  |  |  |  |  |  |  |  |  |  |  |  |  |  |
|  | 217 | 0,50 | 0,50 | 0,50 | 0,50 | 0,50 | 0,50 | 0,50 | 0,55 | 0,50 | 0,50 | 0,50 | 0,58 | 0,50 | 0,50 | 0,44 | 0,50 | 0,50 | 0,50 | 0,50 | 0,50 | 0,50 | 0,50 | 0,50 | 0,50 | 0,50 | 0,50 | 0,50 | 0,50 | 0,50 | 0,50 | 0,50 | 0,50 | 0,50 | 0,50 |
|  | 219 |  |  |  |  |  |  |  |  |  |  |  |  |  |  | **0,03** |  |  |  |  |  |  |  |  |  |  |  |  |  |  |  |  |  |  |  |
|  | 221 |  | 0,50 | 0,50 | 0,50 | 0,48 | 0,44 | 0,50 | 0,45 | 0,25 | 0,50 | 0,50 | 0,42 | 0,50 | 0,50 | 0,47 | 0,50 | 0,50 | 0,50 | 0,50 | 0,50 | 0,50 | 0,50 | 0,50 | 0,50 | 0,50 | 0,50 | 0,50 | 0,46 | 0,50 | 0,50 | 0,50 | 0,50 | 0,50 | 0,50 |
|  | 223 |  |  |  |  |  |  |  |  |  |  |  |  |  |  | **0,03** |  |  |  |  |  |  |  |  |  |  |  |  |  |  |  |  |  |  |  |
|  | 224 | 0,50 |  |  |  | 0,02 | 0,06 |  |  |  |  |  |  |  |  |  |  |  |  |  |  |  |  |  |  |  |  |  | 0,04 |  |  |  |  |  |  |
|  | 225 |  |  |  |  |  |  |  |  | **0,25** |  |  |  |  |  |  |  |  |  |  |  |  |  |  |  |  |  |  |  |  |  |  |  |  |  |
| **Bropap 25444** | 171 |  |  |  |  |  |  |  |  |  |  | 0,03 |  |  |  | 0,03 |  |  |  |  |  |  |  |  |  |  |  |  |  |  |  |  |  |  |  |
|  | 176 |  |  |  |  |  | **0,09** |  |  |  |  |  |  |  |  |  |  |  |  |  |  |  |  |  |  |  |  |  |  |  |  |  |  |  |  |
|  | 177 |  |  |  |  |  |  |  |  |  |  |  |  |  |  | **0,03** |  |  |  |  |  |  |  |  |  |  |  |  |  |  |  |  |  |  |  |
|  | 180 |  |  |  |  |  |  |  |  |  |  |  |  |  |  | **0,03** |  |  |  |  |  |  |  |  |  |  |  |  |  |  |  |  |  |  |  |
|  | 181 |  |  |  |  |  |  |  | **0,10** |  |  |  |  |  |  |  |  |  |  |  |  |  |  |  |  |  |  |  |  |  |  |  |  |  |  |
|  | 184 | 0,50 | 0,50 | 0,50 | 0,50 | 0,50 | 0,41 | 0,50 | 0,45 | 0,50 | 0,50 | 0,47 | 0,50 | 0,50 | 0,50 | 0,47 | 0,50 | 0,50 | 0,50 | 0,50 | 0,50 | 0,50 | 0,50 |  | 0,50 | 0,50 | 0,50 | 0,50 | 0,50 | 0,50 | 0,50 | 0,50 | 0,50 | 0,50 | 0,50 |
|  | 185 |  |  |  |  |  |  |  |  |  |  |  |  |  |  |  |  |  |  |  |  |  |  | **0,17** |  |  |  |  |  |  |  |  |  |  |  |
|  | 186 | 0,50 | 0,50 | 0,50 | 0,50 | 0,50 | 0,50 | 0,50 | 0,40 | 0,50 | 0,50 | 0,47 | 0,50 | 0,50 | 0,50 | 0,39 | 0,50 | 0,50 | 0,50 | 0,50 | 0,50 | 0,50 | 0,50 |  | 0,50 | 0,50 | 0,50 | 0,47 | 0,35 | 0,50 | 0,50 | 0,50 | 0,50 | 0,50 | 0,50 |
|  | 187 |  |  |  |  |  |  |  |  |  |  |  |  |  |  |  |  |  |  |  |  |  |  | **0,50** |  |  |  |  |  |  |  |  |  |  |  |
|  | 188 |  |  |  |  |  |  |  |  |  |  |  |  |  |  |  |  |  |  |  |  |  |  |  |  |  |  | 0,01 | 0,15 |  |  |  |  |  |  |
|  | 189 |  |  |  |  |  |  |  | 0,05 |  |  |  |  |  |  | 0,03 |  |  |  |  |  |  |  | 0,33 |  |  |  |  |  |  |  |  |  |  |  |
|  | 190 |  |  |  |  |  |  |  |  |  |  |  |  |  |  |  |  |  |  |  |  |  |  |  |  |  |  | **0,02** |  |  |  |  |  |  |  |
|  | 198 |  |  |  |  |  |  |  |  |  |  | 0,03 |  |  |  | 0,03 |  |  |  |  |  |  |  |  |  |  |  |  |  |  |  |  |  |  |  |
| **Bropap 26985** | 173 |  |  |  |  |  |  |  |  |  |  |  |  |  |  | **0,06** |  |  |  |  |  |  |  |  |  |  |  |  |  |  |  |  |  |  |  |
|  | 175 |  | 0,50 |  |  |  |  |  |  | 0,25 |  |  |  |  |  |  |  |  |  |  |  |  |  |  |  |  |  |  |  |  |  |  |  |  |  |
|  | 177 | 0,50 |  | 0,50 | 0,50 | 0,50 | 0,50 | 0,50 | 0,50 | 0,25 | 0,50 | 0,50 | 0,50 | 0,50 | 0,50 | 0,44 | 0,50 | 0,50 | 0,50 | 0,50 | 0,50 | 0,50 | 0,50 | 0,50 | 0,50 | 0,50 | 0,50 | 0,50 | 0,50 | 0,50 | 0,50 | 0,50 | 0,50 | 0,50 | 0,50 |
|  | 180 | 0,50 |  |  |  |  |  |  |  |  |  | 0,06 |  |  |  | 0,17 |  |  |  |  |  |  |  |  |  |  |  |  | 0,50 | 0,50 |  |  |  |  | 0,50 |
|  | 182 |  | 0,50 | 0,50 | 0,50 | 0,50 | 0,50 | 0,50 | 0,50 | 0,50 | 0,50 | 0,44 | 0,50 | 0,50 | 0,50 | 0,33 | 0,50 | 0,50 | 0,50 | 0,50 | 0,50 | 0,50 | 0,50 | 0,50 | 0,50 | 0,50 | 0,50 | 0,50 |  |  | 0,50 | 0,50 | 0,50 | 0,50 |  |
| **Bropap 30248** | 87 |  |  |  |  |  |  |  |  | 0,25 |  |  | 0,08 | 0,25 | 0,17 | 0,06 | 0,25 |  |  |  |  |  |  |  |  |  |  |  |  |  |  |  |  |  |  |
|  | 93 | 1,00 | 1,00 | 1,00 | 1,00 | 1,00 | 1,00 | 1,00 | 1,00 | 0,75 | 1,00 | 0,97 | 0,92 | 0,75 | 0,83 | 0,89 | 0,75 | 1,00 | 1,00 | 1,00 | 0,83 | 1,00 | 1,00 | 1,00 | 1,00 | 1,00 | 1,00 | 1,00 | 0,92 | 1,00 | 0,92 | 1,00 | 1,00 | 1,00 | 1,00 |
|  | 95 |  |  |  |  |  |  |  |  |  |  |  |  |  |  |  |  |  |  |  | 0,17 |  |  |  |  |  |  |  | 0,08 |  |  |  |  |  |  |
|  | 97 |  |  |  |  |  |  |  |  |  |  |  |  |  |  | **0,03** |  |  |  |  |  |  |  |  |  |  |  |  |  |  |  |  |  |  |  |
|  | 107 |  |  |  |  |  |  |  |  |  |  | 0,03 |  |  |  | 0,03 |  |  |  |  |  |  |  |  |  |  |  |  |  |  | 0,08 |  |  |  |  |

Private alleles for each locality are marked in **bold type.**
