# Supplementary material for: Human mediated translocation of Pacific paper mulberry [Broussonetia papyrifera (L.) L’Hér. ex Vent. (Moraceae)]: Genetic evidence of dispersal routes in Remote Oceania
Source: PLoS One. 2019 Jun 19;14(6):e0217107. doi: 10.1371/journal.pone.0217107 (PMC6583976; doi:10.1371/journal.pone.0217107)
Supplement: S7 Table — (DOCX) [file pone.0217107.s010.docx]

**S7 Table. Microsatellite genotypes found on different islands and characteristic alleles for each microsatellite marker.**

| **Genotype** | **N of Samples** | **Location** | **Sample source** | **Bro 08** | | **Bro 13** | | **Bro 15** | | **Bropap 2214** | | **Bropap 2801** | | **Bropap 20558** | | **Bropap 25444** | | **Bropap 26985** | | **Bropap 30248** | |
| --- | --- | --- | --- | --- | --- | --- | --- | --- | --- | --- | --- | --- | --- | --- | --- | --- | --- | --- | --- | --- | --- |
| G001 | 1 | China | Contemporary | 210 | 216 | 223 | 229 | 210 | 210 | 236 | 240 | 147 | 147 | 217 | 221 | 183 | 189 | 182 | 182 | 105 | 105 |
| G002 | 1 | China | Contemporary | 196 | 196 | 221 | 227 | 205 | 210 | 230 | 232 | 147 | 149 | 217 | 221 | 170 | 212 | 180 | 184 | 101 | 101 |
| G003 | 1 | China | Contemporary | 188 | 216 | 223 | 226 | 209 | 210 | 234 | 242 | 149 | 149 | 217 | 221 | 178 | 178 | 182 | 182 | 101 | 101 |
| G004 | 1 | China | Contemporary | 198 | 204 | 226 | 226 | 205 | 210 | 230 | 238 | 145 | 149 | 217 | 221 | 182 | 205 | 184 | 184 | 87 | 111 |
| G005 | 1 | China | Herbarium | 204 | 204 | 226 | 226 | 210 | 210 | 223 | 239 | 147 | 149 | 217 | 221 | 177 | 190 | 182 | 182 | 99 | 107 |
| G006 | 1 | Japan | Contemporary | 208 | 208 | 222 | 222 | 210 | 210 | 227 | 231 | 149 | 149 | 217 | 217 | 182 | 182 | 177 | 184 | 112 | 148 |
| G007 | 1 | Japan | Contemporary | 202 | 208 | 222 | 222 | 210 | 210 | 227 | 233 | 149 | 149 | 217 | 217 | 182 | 182 | 177 | 177 | 112 | 112 |
| G008 | 1 | Japan | Contemporary | 208 | 208 | 222 | 222 | 210 | 210 | 231 | 255 | 149 | 149 | 217 | 220 | 182 | 184 | 177 | 184 | 100 | 114 |
| G009 | 1 | Japan | Contemporary | 208 | 208 | 222 | 222 | 210 | 210 | 227 | 231 | 149 | 149 | 217 | 217 | 182 | 182 | 177 | 177 | 100 | 100 |
| G010 | 1 | Japan | Contemporary | 202 | 208 | 222 | 222 | 210 | 210 | 231 | 231 | 149 | 149 | 217 | 217 | 184 | 184 | 177 | 184 | 112 | 112 |
| G011 | 1 | New Zealand | Herbarium | 206 | 208 | 220 | 227 | 205 | 210 | 231 | 243 | 149 | 154 | 217 | 220 | 183 | 206 | 184 | 184 | 101 | 123 |
| G012 | 1 | New Zealand | Herbarium | 208 | 208 | 226 | 226 | 202 | 202 | 239 | 241 | 149 | 149 | 221 | 221 | 175 | 216 | 177 | 190 | 93 | 97 |
| G013 | 1 | Santiago | Contemporary | 196 | 208 | 227 | 227 | 205 | 205 | 225 | 247 | 149 | 153 | 217 | 220 | 178 | 178 | 182 | 182 | 95 | 123 |
| G014 | 1 | Santiago | Herbarium | 208 | 216 | 227 | 227 | 205 | 205 | 247 | 247 | 154 | 154 | 217 | 220 | 204 | 206 | 182 | 182 | 123 | 123 |
| G015 | 1 | Solomon Is. | Herbarium | 194 | 206 | 221 | 222 | 210 | 210 | 231 | 239 | 149 | 149 | 217 | 223 | 177 | 179 | 182 | 182 | 123 | 123 |
| G016 | 1 | Solomon Is. | Herbarium | 182 | 194 | 221 | 222 | 210 | 210 | 231 | 231 | 140 | 149 | 204 | 217 | 178 | 178 | 173 | 182 | 116 | 123 |
| G017 | 1 | Solomon Is. | Herbarium | 182 | 194 | 221 | 222 | 210 | 210 | 231 | 239 | 147 | 149 | 211 | 223 | 170 | 178 | 173 | 182 | 123 | 136 |
| G018 | 1 | Taiwan | Contemporary | 192 | 220 | 223 | 224 | 214 | 214 | 243 | 245 | 149 | 149 | 221 | 221 | 173 | 173 | 182 | 182 | 91 | 91 |
| G019 | 1 | Taiwan | Contemporary | 188 | 212 | 226 | 226 | 210 | 210 | 241 | 247 | 149 | 149 | 217 | 217 | 175 | 175 | 177 | 190 | 107 | 107 |
| G020 | 1 | Taiwan | Contemporary | 192 | 192 | 223 | 223 | 210 | 214 | 229 | 241 | 159 | 168 | 217 | 217 | 192 | 211 | 177 | 184 | 89 | 89 |
| G021 | 1 | Taiwan | Contemporary | 194 | 202 | 226 | 226 | 210 | 210 | 229 | 229 | 149 | 149 | 221 | 221 | 197 | 203 | 182 | 182 | 93 | 93 |
| G022 | 1 | Taiwan | Contemporary | 200 | 218 | 223 | 226 | 202 | 210 | 229 | 251 | 149 | 159 | 217 | 221 | 203 | 205 | 177 | 177 | 89 | 93 |
| G023 | 1 | Taiwan | Contemporary | 200 | 208 | 223 | 226 | 214 | 214 | 227 | 241 | 149 | 149 | 217 | 221 | 173 | 175 | 177 | 190 | 93 | 93 |
| G024 | 1 | Taiwan | Contemporary | 188 | 192 | 226 | 226 | 214 | 214 | 243 | 243 | 149 | 149 | 217 | 221 | 173 | 173 | 177 | 190 | 91 | 91 |
| G025 | 1 | Taiwan | Contemporary | 192 | 206 | 223 | 226 | 210 | 210 | 233 | 241 | 149 | 149 | 221 | 221 | 173 | 173 | 182 | 190 | 91 | 91 |
| G026 | 1 | Taiwan | Contemporary | 202 | 216 | 224 | 226 | 202 | 210 | 239 | 247 | 149 | 170 | 217 | 221 | 173 | 173 | 177 | 184 | 91 | 91 |
| G027 | 1 | Taiwan | Contemporary | 206 | 206 | 223 | 226 | 210 | 214 | 249 | 249 | 159 | 170 | 217 | 221 | 157 | 177 | 177 | 190 | 91 | 91 |
| G028 | 2 | Taiwan | Contemporary | 200 | 212 | 223 | 224 | 210 | 210 | 241 | 245 | 149 | 159 | 217 | 217 | 173 | 175 | 177 | 190 | 95 | 97 |
| G029 | 1 | Taiwan | Contemporary | 208 | 214 | 226 | 226 | 210 | 214 | 241 | 249 | 149 | 170 | 218 | 221 | 173 | 173 | 177 | 182 | 93 | 95 |
| G030 | 1 | Taiwan | Contemporary | 192 | 214 | 224 | 224 | 202 | 214 | 241 | 245 | 149 | 151 | 221 | 221 | 173 | 173 | 182 | 182 | 91 | 93 |
| G031 | 1 | New Guinea | Herbarium | 202 | 206 | 226 | 226 | 210 | 214 | 241 | 241 | 149 | 170 | 217 | 221 | 184 | 186 | 177 | 182 | 93 | 93 |
| G032 | 1 | New Guinea | Herbarium | 202 | 206 | 226 | 226 | 210 | 214 | 241 | 251 | 149 | 170 | 217 | 221 | 184 | 186 | 177 | 182 | 93 | 93 |
| G033 | 1 | Hawaii (f) | Contemporary | 202 | 206 | 226 | 226 | 210 | 221 | 241 | 249 | 149 | 170 | 217 | 221 | 171 | 198 | 177 | 182 | 93 | 93 |
| G034 | 1 | Taiwan | Contemporary | 200 | 206 | 223 | 226 | 210 | 210 | 229 | 237 | 149 | 168 | 221 | 221 | 173 | 198 | 180 | 182 | 95 | 95 |
| G035 | 1 | Taiwan | Contemporary | 200 | 202 | 223 | 226 | 214 | 214 | 245 | 247 | 149 | 149 | 221 | 221 | 173 | 173 | 182 | 184 | 106 | 109 |
| G036 | 1 | Taiwan | Contemporary | 200 | 200 | 226 | 226 | 214 | 214 | 239 | 243 | 149 | 159 | 217 | 221 | 175 | 192 | 182 | 182 | 97 | 97 |
| G037 | 1 | Taiwan | Contemporary | 202 | 210 | 223 | 226 | 210 | 210 | 228 | 228 | 157 | 159 | 217 | 217 | 173 | 186 | 177 | 177 | 83 | 83 |
| G038 | 1 | Taiwan | Contemporary | 188 | 196 | 226 | 226 | 210 | 214 | 227 | 249 | 159 | 170 | 217 | 221 | 173 | 207 | 177 | 184 | 83 | 91 |
| G039 | 1 | Vietnam | Contemporary | 196 | 196 | 220 | 221 | 210 | 210 | 229 | 245 | 149 | 151 | 217 | 223 | 176 | 178 | 184 | 184 | 144 | 144 |

**Continuation II. S7 Table**

| **Genotype** | **N of Samples** | **Location** | **Sample source** | **Bro 08** | | **Bro 13** | | **Bro 15** | | **Bropap 2214** | | **Bropap 2801** | | **Bropap 20558** | | **Bropap 25444** | | **Bropap 26985** | | **Bropap 30248** | |
| --- | --- | --- | --- | --- | --- | --- | --- | --- | --- | --- | --- | --- | --- | --- | --- | --- | --- | --- | --- | --- | --- |
| G040 | 1 | Vietnam | Contemporary | 194 | 194 | 221 | 226 | 147 | 149 | 229 | 239 | 147 | 149 | 217 | 217 | 207 | 225 | 182 | 182 | 99 | 99 |
| G041 | 1 | Vietnam | Contemporary | 194 | 200 | 220 | 221 | 206 | 206 | 220 | 229 | 147 | 149 | 220 | 223 | 177 | 179 | 182 | 182 | 103 | 132 |
| G042 | 1 | Vietnam | Contemporary | 186 | 194 | 220 | 226 | 210 | 210 | 229 | 231 | 147 | 149 | 221 | 223 | 173 | 175 | 182 | 182 | 123 | 156 |
| G043 | 1 | Vietnam | Contemporary | 194 | 194 | 221 | 223 | 210 | 210 | 229 | 239 | 149 | 149 | 221 | 221 | 177 | 181 | 182 | 182 | 134 | 134 |
| G044 | 1 | Fiji | Contemporary | 202 | 206 | 226 | 226 | 210 | 214 | 243 | 251 | 149 | 172 | 217 | 224 | 184 | 186 | 177 | 182 | 93 | 93 |
| G045 | 1 | Fiji | Contemporary | 202 | 206 | 226 | 226 | 210 | 214 | 241 | 253 | 149 | 170 | 217 | 221 | 184 | 186 | 177 | 182 | 93 | 93 |
| G046 | 1 | Fiji | Contemporary | 202 | 206 | 226 | 226 | 210 | 214 | 247 | 251 | 149 | 170 | 217 | 221 | 176 | 186 | 177 | 182 | 93 | 93 |
| G047 | 2 | Fiji | Contemporary | 202 | 206 | 226 | 226 | 210 | 214 | 247 | 251 | 149 | 170 | 217 | 224 | 176 | 186 | 177 | 182 | 93 | 93 |
| G048 | 1 | Fiji | Contemporary | 202 | 206 | 226 | 226 | 210 | 214 | 241 | 249 | 149 | 170 | 217 | 221 | 184 | 186 | 177 | 182 | 93 | 93 |
| G048 | 5 | Pitcairn | Contemporary and Herbarium | 202 | 206 | 226 | 226 | 210 | 214 | 241 | 249 | 149 | 170 | 217 | 221 | 184 | 186 | 177 | 182 | 93 | 93 |
| G048 | 1 | Tonga | Contemporary | 202 | 206 | 226 | 226 | 210 | 214 | 241 | 249 | 149 | 170 | 217 | 221 | 184 | 186 | 177 | 182 | 93 | 93 |
| G049 | 68 | Fiji | Contemporary | 202 | 206 | 226 | 226 | 210 | 214 | 241 | 249 | 149 | 172 | 217 | 221 | 184 | 186 | 177 | 182 | 93 | 93 |
| G049 | 1 | Futuna | Herbarium | 202 | 206 | 226 | 226 | 210 | 214 | 241 | 249 | 149 | 172 | 217 | 221 | 184 | 186 | 177 | 182 | 93 | 93 |
| G049 | 36 | Tonga | Contemporary | 202 | 206 | 226 | 226 | 210 | 214 | 241 | 249 | 149 | 172 | 217 | 221 | 184 | 186 | 177 | 182 | 93 | 93 |
| G050 | 1 | Fiji | Herbarium | 202 | 206 | 226 | 226 | 210 | 214 | 243 | 251 | 149 | 170 | 217 | 225 | 184 | 186 | 175 | 182 | 87 | 93 |
| G051 | 1 | Fiji | Herbarium | 202 | 206 | 226 | 226 | 210 | 214 | 239 | 241 | 149 | 172 | 217 | 221 | 184 | 189 | 177 | 182 | 93 | 93 |
| G052 | 1 | Fiji | Herbarium | 202 | 206 | 226 | 226 | 210 | 214 | 251 | 251 | 149 | 172 | 217 | 217 | 181 | 181 | 177 | 182 | 93 | 93 |
| G053 | 1 | Fiji | Herbarium | 202 | 206 | 226 | 226 | 210 | 214 | 235 | 241 | 149 | 172 | 217 | 221 | 184 | 186 | 177 | 182 | 93 | 93 |
| G054 | 1 | Samoa | Contemporary | 202 | 206 | 226 | 226 | 210 | 214 | 241 | 249 | 149 | 172 | 217 | 221 | 184 | 186 | 177 | 180 | 95 | 95 |
| G055 | 2 | Samoa | Contemporary | 202 | 206 | 226 | 226 | 210 | 214 | 241 | 251 | 149 | 172 | 217 | 221 | 184 | 186 | 177 | 180 | 93 | 93 |
| G056 | 1 | Samoa | Herbarium | 202 | 206 | 226 | 226 | 210 | 214 | 243 | 251 | 149 | 160 | 217 | 224 | 184 | 186 | 177 | 180 | 93 | 93 |
| G057 | 4 | Samoa | Contemporary | 202 | 206 | 226 | 226 | 210 | 214 | 241 | 249 | 149 | 172 | 217 | 221 | 184 | 188 | 177 | 180 | 93 | 93 |
| G058 | 10 | Samoa | Contemporary | 202 | 206 | 226 | 226 | 210 | 214 | 241 | 249 | 149 | 172 | 217 | 221 | 184 | 186 | 177 | 180 | 93 | 93 |
| G058 | 13 | Wallis | Contemporary | 202 | 206 | 226 | 226 | 210 | 214 | 241 | 249 | 149 | 172 | 217 | 221 | 184 | 186 | 177 | 180 | 93 | 93 |
| G059 | 1 | Samoa | Herbarium | 202 | 206 | 226 | 226 | 210 | 214 | 243 | 251 | 149 | 172 | 217 | 224 | 184 | 186 | 177 | 180 | 93 | 93 |
| G060 | 1 | Tonga | Contemporary | 202 | 210 | 226 | 226 | 210 | 214 | 241 | 249 | 149 | 172 | 217 | 221 | 184 | 186 | 177 | 182 | 93 | 93 |
| G061 | 1 | Tonga | Contemporary | 204 | 210 | 226 | 226 | 210 | 214 | 241 | 249 | 149 | 172 | 217 | 221 | 184 | 186 | 177 | 182 | 93 | 93 |
| G062 | 1 | Tonga | Contemporary | 198 | 206 | 226 | 226 | 210 | 214 | 241 | 249 | 149 | 172 | 217 | 221 | 184 | 186 | 177 | 182 | 93 | 93 |
| G063 | 1 | Austral Isl. | Herbarium | 202 | 206 | 226 | 226 | 210 | 221 | 241 | 249 | 149 | 170 | 217 | 221 | 184 | 186 | 177 | 182 | 93 | 93 |
| G063 | 20 | Hawaii (f) | Contemporary and Herbarium | 202 | 206 | 226 | 226 | 210 | 221 | 241 | 249 | 149 | 170 | 217 | 221 | 184 | 186 | 177 | 182 | 93 | 93 |
| G063 | 10 | Marquesas | Contemporary and Herbarium | 202 | 206 | 226 | 226 | 210 | 221 | 241 | 249 | 149 | 170 | 217 | 221 | 184 | 186 | 177 | 182 | 93 | 93 |
| G063 | 1 | Tonga | Contemporary | 202 | 206 | 226 | 226 | 210 | 221 | 241 | 249 | 149 | 170 | 217 | 221 | 184 | 186 | 177 | 182 | 93 | 93 |
| G064 | 1 | Hawaii (f) | Contemporary | 204 | 206 | 226 | 226 | 210 | 214 | 241 | 249 | 149 | 172 | 217 | 221 | 184 | 186 | 177 | 182 | 93 | 93 |
| G064 | 10 | Tonga | Contemporary and Herbarium | 204 | 206 | 226 | 226 | 210 | 214 | 241 | 249 | 149 | 172 | 217 | 221 | 184 | 186 | 177 | 182 | 93 | 93 |
| G065 | 3 | Rapa | Contemporary and Herbarium | 202 | 206 | 226 | 226 | 210 | 221 | 241 | 249 | 149 | 170 | 217 | 221 | 184 | 186 | 175 | 182 | 93 | 93 |
| G066 | 4 | Rapa | Herbarium | 202 | 220 | 226 | 226 | 210 | 221 | 241 | 249 | 149 | 170 | 217 | 221 | 184 | 186 | 175 | 182 | 93 | 93 |
| G067 | 1 | Cook Is. | Herbarium | 202 | 206 | 226 | 226 | 206 | 210 | 241 | 249 | 149 | 170 | 217 | 221 | 184 | 186 | 177 | 182 | 93 | 93 |
| G068 | 1 | Marquesas | Contemporary | 202 | 202 | 226 | 226 | 210 | 221 | 241 | 249 | 149 | 170 | 217 | 221 | 184 | 186 | 177 | 182 | 93 | 95 |
| G069 | 1 | Marquesas | Contemporary | 202 | 206 | 226 | 226 | 210 | 214 | 243 | 253 | 149 | 170 | 217 | 221 | 184 | 186 | 177 | 182 | 93 | 93 |
| G069 | 1 | Tahiti | Contemporary | 202 | 206 | 226 | 226 | 210 | 214 | 243 | 253 | 149 | 170 | 217 | 221 | 184 | 186 | 177 | 182 | 93 | 93 |

**Continuation III. S7 Table**

| **Genotype** | **N of Samples** | **Location** | **Sample source** | **Bro 08** | | **Bro 13** | | **Bro 15** | | **Bropap 2214** | | **Bropap 2801** | | **Bropap 20558** | | **Bropap 25444** | | **Bropap 26985** | | **Bropap 30248** | |
| --- | --- | --- | --- | --- | --- | --- | --- | --- | --- | --- | --- | --- | --- | --- | --- | --- | --- | --- | --- | --- | --- |
| G070 | 2 | Marquesas | Contemporary | 202 | 206 | 226 | 226 | 210 | 214 | 243 | 249 | 149 | 170 | 217 | 221 | 184 | 186 | 177 | 182 | 93 | 93 |
| G070 | 31 | Rapa Nui | Contemporary and Herbarium | 202 | 206 | 226 | 226 | 210 | 214 | 243 | 249 | 149 | 170 | 217 | 221 | 184 | 186 | 177 | 182 | 93 | 93 |
| G071 | 2 | Niue | Herbarium | 202 | 206 | 226 | 226 | 210 | 221 | 241 | 249 | 149 | 172 | 217 | 221 | 184 | 186 | 177 | 182 | 93 | 93 |
| G071 | 4 | Tahiti | Contemporary | 202 | 206 | 226 | 226 | 210 | 221 | 241 | 249 | 149 | 172 | 217 | 221 | 184 | 186 | 177 | 182 | 93 | 93 |
| G072 | 1 | New Caledonia | Contemporary | 202 | 206 | 226 | 226 | 210 | 214 | 243 | 249 | 149 | 180 | 217 | 221 | 185 | 187 | 177 | 182 | 93 | 93 |
| G073 | 2 | New Caledonia | Contemporary | 202 | 206 | 226 | 226 | 210 | 214 | 243 | 249 | 149 | 180 | 217 | 221 | 187 | 189 | 177 | 182 | 93 | 93 |
| G074 | 1 | Marquesas | Contemporary | 202 | 208 | 226 | 226 | 210 | 214 | 243 | 249 | 149 | 170 | 217 | 221 | 184 | 186 | 177 | 182 | 93 | 93 |
| G075 | 1 | Tahiti | Contemporary | 202 | 206 | 226 | 226 | 210 | 214 | 243 | 249 | 149 | 170 | 217 | 221 | 184 | 186 | 177 | 182 | 93 | 107 |
| G076 | 1 | Rapa | Contemporary | 202 | 206 | 226 | 226 | 210 | 221 | 249 | 249 | 149 | 170 | 217 | 221 | 184 | 186 | 175 | 182 | 93 | 93 |
| G077 | 1 | Rapa Nui | Contemporary | 202 | 210 | 226 | 226 | 210 | 214 | 243 | 249 | 149 | 170 | 217 | 221 | 184 | 186 | 177 | 182 | 93 | 93 |
| G078 | 3 | Rapa Nui | Contemporary and Herbarium | 202 | 206 | 226 | 226 | 210 | 214 | 247 | 251 | 149 | 170 | 217 | 221 | 184 | 186 | 177 | 182 | 93 | 93 |
| G079 | 3 | Rapa Nui | Contemporary | 202 | 206 | 226 | 226 | 210 | 214 | 243 | 249 | 149 | 170 | 217 | 221 | 184 | 190 | 177 | 182 | 93 | 93 |
| G080 | 3 | Rapa Nui | Contemporary | 204 | 208 | 226 | 226 | 210 | 214 | 243 | 249 | 149 | 170 | 217 | 221 | 184 | 186 | 177 | 182 | 93 | 93 |
| G081 | 24 | Rapa Nui | Contemporary | 202 | 206 | 226 | 226 | 210 | 214 | 243 | 251 | 149 | 170 | 217 | 221 | 184 | 186 | 177 | 182 | 93 | 93 |
| G082 | 1 | Hawaii (f) | Contemporary | 206 | 208 | 226 | 226 | 210 | 214 | 241 | 249 | 149 | 159 | 217 | 221 | 171 | 198 | 177 | 180 | 93 | 93 |
| G083 | 1 | Hawaii (f) | Contemporary | 202 | 206 | 226 | 226 | 210 | 214 | 241 | 251 | 149 | 159 | 217 | 221 | 184 | 186 | 177 | 180 | 93 | 93 |
| G084 | 1 | Hawaii (f) | Contemporary | 202 | 206 | 226 | 226 | 210 | 214 | 243 | 249 | 149 | 172 | 217 | 221 | 184 | 186 | 177 | 182 | 93 | 93 |
| G084 | 2 | Tonga | Contemporary | 202 | 206 | 226 | 226 | 210 | 214 | 243 | 249 | 149 | 172 | 217 | 221 | 184 | 186 | 177 | 182 | 93 | 93 |
| G085 | 1 | Hawaii (f) | Herbarium | 202 | 206 | 226 | 226 | 210 | 221 | 241 | 251 | 149 | 170 | 217 | 221 | 184 | 186 | 177 | 182 | 93 | 107 |
| G086 | 1 | Hawaii | Herbarium | 202 | 209 | 226 | 226 | 210 | 228 | 239 | 241 | 149 | 170 | 219 | 223 | 180 | 186 | 177 | 182 | 93 | 93 |
| G087 | 1 | Hawaii | Herbarium | 202 | 206 | 226 | 226 | 210 | 221 | 241 | 249 | 149 | 170 | 217 | 221 | 184 | 186 | 173 | 182 | 87 | 93 |
| G088 | 6 | Hawaii (f) | Contemporary | 206 | 208 | 226 | 226 | 210 | 214 | 241 | 249 | 149 | 159 | 217 | 221 | 184 | 186 | 177 | 180 | 93 | 93 |
| G089 | 1 | Hawaii | Herbarium | 202 | 208 | 222 | 222 | 210 | 210 | 219 | 229 | 145 | 177 | 206 | 217 | 184 | 184 | 173 | 182 | 93 | 97 |
| G090 | 3 | Hawaii | Herbarium | 202 | 206 | 226 | 226 | 210 | 221 | 241 | 251 | 149 | 170 | 217 | 221 | 184 | 186 | 177 | 182 | 93 | 93 |
| G091 | 1 | Hawaii | Herbarium | 202 | 206 | 226 | 226 | 210 | 221 | 241 | 249 | 149 | 170 | 217 | 221 | 184 | 189 | 177 | 182 | 93 | 93 |
| G092 | 1 | Hawaii (f) | Herbarium | 202 | 206 | 226 | 226 | 210 | 221 | 241 | 249 | 149 | 170 | 217 | 221 | 184 | 186 | 177 | 182 | 93 | 107 |
| G093 | 1 | Hawaii (f) | Herbarium | 202 | 202 | 226 | 226 | 210 | 221 | 236 | 244 | 170 | 170 | 221 | 221 | 177 | 184 | 177 | 182 | 93 | 93 |
| G094 | 1 | Hawaii (f) | Herbarium | 202 | 206 | 226 | 226 | 210 | 221 | 241 | 241 | 149 | 170 | 217 | 221 | 184 | 186 | 177 | 182 | 93 | 93 |
| G095 | 1 | Hawaii (f) | Herbarium | 202 | 206 | 226 | 226 | 210 | 221 | 241 | 253 | 149 | 170 | 217 | 221 | 184 | 186 | 177 | 182 | 93 | 93 |
| G096 | 1 | Hawaii | Herbarium | 182 | 206 | 226 | 226 | 210 | 221 | 241 | 249 | 149 | 170 | 217 | 221 | 184 | 186 | 177 | 182 | 87 | 93 |
| G097 | 1 | Hawaii (f) | Herbarium | 202 | 202 | 226 | 226 | 210 | 210 | 241 | 249 | 149 | 149 | 217 | 217 | 184 | 186 | 177 | 182 | 93 | 93 |
| G098 | 4 | Hawaii | Herbarium | 202 | 206 | 226 | 226 | 210 | 221 | 241 | 249 | 149 | 170 | 217 | 221 | 184 | 186 | 177 | 182 | 87 | 93 |
| G099 | 1 | Hawaii (m) | Contemporary | 202 | 206 | 220 | 226 | 213 | 213 | 231 | 237 | 139 | 147 | 220 | 220 | 184 | 186 | 182 | 182 | 89 | 112 |
| G100 | 2 | Hawaii (m) | Contemporary | 202 | 206 | 220 | 226 | 213 | 213 | 231 | 237 | 139 | 147 | 220 | 220 | 171 | 198 | 182 | 182 | 89 | 112 |
| G101 | 1 | Hawaii (m) | Contemporary | 202 | 206 | 221 | 226 | 210 | 213 | 231 | 235 | 139 | 147 | 220 | 220 | 184 | 186 | 182 | 182 | 89 | 89 |
| G102 | 7 | Hawaii (m) | Contemporary | 202 | 206 | 221 | 226 | 210 | 213 | 231 | 235 | 139 | 147 | 220 | 220 | 171 | 198 | 182 | 182 | 89 | 89 |
| G103 | 7 | Hawaii (m) | Contemporary | 202 | 206 | 220 | 226 | 213 | 213 | 231 | 237 | 139 | 147 | 220 | 220 | 171 | 198 | 182 | 182 | 89 | 125 |
| G104 | 1 | Marquesas | Herbarium | 192 | 202 | 226 | 226 | 210 | 221 | 241 | 251 | 149 | 170 | 217 | 221 | 184 | 186 | 177 | 182 | 93 | 93 |
