# Supplementary material for: Human mediated translocation of Pacific paper mulberry [Broussonetia papyrifera (L.) L’Hér. ex Vent. (Moraceae)]: Genetic evidence of dispersal routes in Remote Oceania
Source: PLoS One. 2019 Jun 19;14(6):e0217107. doi: 10.1371/journal.pone.0217107 (PMC6583976; doi:10.1371/journal.pone.0217107)
Supplement: S1 Text — (DOCX) [file pone.0217107.s011.docx]

**SUPPLEMENTARY TEXT**

**SAMPLING PERMITS:**

**Contemporary leaf samples**:

Collecting permits were issued by CONAF (Corporación Nacional Forestal), Chile for collecting samples within the National Park on Easter Island. Samples from French Polynesia were collected under a permit issued by the Head of the Department, Délégation à la Recherche of French Polynesia in 2008 in Papeete, Tahiti. Samples from Rapa were sent by J.Y Meyer from the Delegation de la Rechérche, Tahiti, Polynesie Française. A permit to collect samples in Wallis was issued by the Service Territorial, Affaires Culturelles of Wallis and Futuna in 2013. Amy Greenwell Botanic Garden, Hawaii, sent samples collected by their staff. In addition, all samples taken in private gardens from Rapa Nui (Easter Island) and Samoa and village fields in Tonga were taken with permission of the owners in every case. In Fiji samples were collected after permission of village chiefs and acknowledged by the Fiji National Biodiversity Strategy Action Plan, Ministry of Local Government, Urban Development and Environment, Department of Environment, Suva, Fiji. Samples from Pitcairn, New Caledonia and Japan were sent to us by local residents. Fresh leaf samples from Hawaii were given to us by local Hawaiian kapa makers from their own plantations. Samples from Taiwan and China were provided by one of the co-authors. Samples from Vietnam were sent to us. Samples from Santiago were collected by us from a tree on the street.

**Herbarium samples**:

Herbarium sampling was approved by the respective curators: Barbara Kennedy, curator of the Herbarium Pacificum (BISH) for samples from Pitcairn, Hawaii, Rapa, Marquesas, Austral Islands, American Samoa, Cook Islands, New Guinea, Tahiti, Fiji, Tonga, Niue and Solomon Islands accessions; Ewen Cameron, curator of the Auckland Museum Herbarium (AK), for samples from New Zealand, New Guinea and Solomon Islands accessions; Gloria Rojas, curator of the Herbarium from the Museo Nacional de Historia Natural, Santiago, Chile (SGO) for samples from accessions from Rapa Nui, mainland Chile and China.
